# Impact of Infant and Young Child Feeding (IYCF) Nutrition Interventions on Breastfeeding Practices, Growth and Mortality in Low- and Middle-Income Countries: Systematic Review

**DOI:** 10.3390/nu12030722

**Published:** 2020-03-10

**Authors:** Zohra S. Lassi, Fahad Rind, Omar Irfan, Rabia Hadi, Jai K. Das, Zulfiqar A. Bhutta

**Affiliations:** 1 Robinson Research Institute, University of Adelaide, Adelaide 5005, Australia; 2 Division of Women and Child Health, Aga Khan University Hospital, Karachi 74800, Pakistan; dr.fahadrind@gmail.com (F.R.); zulfiqar.bhutta@sickkids.ca (Z.A.B.); 3 Centre for Global Child Health, The Hospital for Sick Children, Toronto, ON M5G 0A4, Canada; omarirfan1@hotmail.com; 4 Center of Excellence in Women and Child Health, The Aga Khan University, Karachi 74800, Pakistan; hadirabia@gmail.com (R.H.); jai.das@aku.edu (J.K.D.)

**Keywords:** breastfeeding, infants, growth, mortality, LMIC, young child, complementary feeding, supplementary feeding, malnutrition

## Abstract

Undernutrition is associated with 45% of total infant deaths, totalling 2.7 million globally per year. The vast majority of the burden is felt in low- and middle-income countries (LMICs). This review aims to assess the effectiveness of infant and young child feeding (IYCF) interventions. We searched multiple databases including Cochrane Controlled Trials Register (CENTRAL), MEDLINE, EMBASE. Title/abstract screening and full-text screening and data extraction filtered 77 studies for inclusion. Breastfeeding education interventions (*n* = 38) showed 20% increase in rates of early initiation of breastfeeding, 102% increase in exclusive breastfeeding (EBF) at 3 months and 53% increase in EBF at 6 months and 24% decreases in diarrheal diseases. Complementary feeding education intervention (n=12) showed a 0.41 standard deviation (SD) increase in WAZ, and 0.25 SD in HAZ in food secure setting. Complementary food provision with or without education (n=17) showed a 0.14 SD increase in HAZ and 36% decrease in stunting. Supplementary food interventions (n=12) showed a significant 0.15 SD increase in WHZ. Subgroup analyses showed healthcare professional led interventions were largely more effective, especially on breastfeeding outcomes. We believe this is a comprehensive review of the existing literature on IYCF studies in LMICs. Though breastfeeding education is well supported in its effectiveness on breastfeeding practices, limited evidence exists for growth outcomes. Supplementation interventions seem to have better effects at improving growth. However, more research is required to reach more substantial conclusions.

## 1. Introduction

Undernutrition is associated with 2.7 million child deaths worldwide which makes up 45% of all infant mortality, according to the World Health Organization [1]. Lower- and middle-income countries (LMICs) take the overwhelming brunt of the tragedy, accounting for 99% of such instances of infant mortality in Sub-Saharan Africa and South Asia [1]. It is estimated that 250 million children under the age of five are at risk of sub-optimal development and stunting [1]. Infant malnutrition has been associated with increased severity and frequency of infections, raising energy requirements, while reducing appetite and nutrition absorption [2]. This is ultimately increasing the risk of death [3]. Another effect of malnutrition is cognitive development which can affect school performance and has negative effects on long term careers [3]. The cognitive development has been known to have economic effects linked to productivity and gross domestic product (GDP) of the country. Stunting has been linked to cognitive delays and increased mortality [4], while wasting contributes to stunting itself [5].

Acute malnutrition is estimated to affect 51.5 million children under five years, contributing to 12.6% of under-five mortality worldwide [6]. Severe acute malnutrition (SAM) is defined as a weight-for-height at −3 Z-scores of median WHO growth standards with visible severe wasting and/or presence of nutritional edema [1]. Moderate acute malnutrition (MAM) is defined as a less severe form for SAM, weight-for-height between −2 and −3 Z-scores of median WHO growth standards [1]. In 2011, 16% of infants had a weight-for-age Z-scores (WAZ) below −2 Z-score [7].

Breastfeeding and complementary foods make up most of the nutrition in early life. Breastfeeding has been proven to be the most optimal nutrition for infants. This is due to immunologic, hormonal and growth advantages which make for a protective and optimal nutrition for a child [8]. Immunologic protection is provided by antibodies present in the breast milk [8] which can promote quick recovery from illness [9]. Oligosaccharides inhibit the binding of pathogens and toxins to host receptors, thus preventing infection [10]. In addition to the obvious benefit of immediate energy, breast milk has been shown to improve neurodevelopment, namely improve cognition [8]. The participating women also benefit from reduced rates of ovarian and breast cancer and diabetes due to breastfeeding [11,12]. It is, therefore, very evident, that breastfeeding is almost essential for maternal and infant health benefits.

Physiologically, breast milk can only provide nutrition till a certain age at which point the infants’ diet requires the addition of solid food. This point comes at six months age. It then becomes necessary to increase the nutrient intake to ensure optimal growth and development. It is important to note that early nutrition can have irreparable consequences as after the age of two, stunting and other growth deficiencies can be very difficult to reverse [13]. Currently, the rates of continued breastfeeding drop from 74% at 12 months to 46% at 24 months of age [14]. One third of children aged 4 to 5 months are incorrectly already on complementary feeding while conversely one fifth of 10–11-month-olds are exclusively on breastfeeding. Additionally, it was found that nearly one third of children ages 6–23 months were receiving a minimally diverse diet [14]. This outlines the importance and need of providing infants with required nutrition through proper complementary feeding practices. Complementary foods can include lipid-based, ready-to-use therapeutic foods, grain and starchy foods, meat, fish, vegetables, eggs and even some traditional foods such as *khichuri* which is a rich mix of lentils, rice and vegetables in the subcontinent. Well-known supplementary foods include the Plumpy’doz, rice and lentil packages and wheat soy blends [15]. Such forms of supplementation can be particularly important and even necessary in LMICs where monetary impossibilities can impede nutritional potential.

A holistic review of the infant and young child feeding (IYCF) interventions would be valuable as most reviews are limited to a single study design type, setting, socioeconomic setting, single interventions, timing and duration of interventions etc. This systematic review could potentially play a large role in policy-making to maximally improve IYCF outcomes. As a result, this review aims to systematically review the current evidence body on the effectiveness of IYCF interventions and programs and their effect on breastfeeding and nutritional outcomes.

In addition, this review aims to study the effectiveness of IYCF interventions on good breastfeeding practice and growth outcomes.

## 2. Materials and Methods

The original protocol for this systematic review was published on 30 December 2018 [16].

### 2.1. Objectives

The overall objectives are to assess the effectiveness of the following on child health and nutritional status:

- Interventions to promote early and exclusive breastfeeding;
- Interventions to promote continued breastfeeding;
- Interventions to promote appropriate complementary feeding (education or provision of complementary food) during infancy and childhood;
- Effectiveness of community-based interventions to prevent moderate and severe acute malnutrition.

#### 2.1.1. Types of Studies and Participants

We included primary studies, including large-scale program evaluations to assess the efficacy and/or effectiveness of interventions. We included randomized controlled trials (RCTs) randomized at the individual or cluster level (i.e., in cluster randomized controlled trial), cross-over study designs. We also included non-randomized studies such as quasi-experimental designs, including controlled before-after studies (CBA), interrupted time series (ITS) studies, natural experiments and regression discontinuity designs. Non-controlled pre-post studies were not included.

#### 2.1.2. Types of Participants

Our target population of study was mothers/nursing mothers of children under 2 years of age living in lower middle-income countries (LMICs) of reproductive age, regardless of comorbid and general health status. Participants could be pregnant while interventions were applied. We also included studies in which only a portion of the study sample fit our inclusion criteria, provided they were compared independently to a control group and analyzed independently. Conversely, if arms of an included study did not fit our inclusion criteria, those arms of interventions were not included in our analysis.

#### 2.1.3. Types of Interventions

This review focused on the effects of the following interventions:

1. Interventions designed to promote early initiation, exclusive and continued breastfeeding, such as education and support of early and exclusive breastfeeding practices.
2. Interventions to promote dietary diversification and appropriate complementary feeding. These will be divided in interventions that provide only education and interventions in which the provision of complementary food for healthy individuals takes place regardless of education.
3. Interventions to prevent moderate and severe acute malnutrition, such as educative interventions and supplementary food for children suffering from MAM or SAM.

Interventions were supervised by healthcare professionals, community health workers and through telephone network platforms e.g., text messages, phone apps. Interventions were compared to control groups receiving no interventions or standard of care.

#### 2.1.4. Types of Outcome Measures

Primary outcomes: Early initiation of breastfeeding (within an hour of birth), exclusive breastfeeding (EBF) at 3 and 6 months of age, infant growth (weight gain (kg), height gain (cm), Z-scores for height-for-age (HAZ), weight-for-age (WAZ), weight-for-height (WHZ), stunting (HAZ < 2), wasting (WAZ < 2), underweight (WHZ < 2)). Our secondary outcomes included neonatal mortality (death from all causes within a month of birth per total live births), infant mortality (death from all causes within 12 months of age per total live births), neonatal sepsis (the proportion of neonates dying due to possible serious bacterial infections among all neonates), incidence of acute respiratory infections (ARI), incidence of diarrheal illness, any adverse events including gastrointestinal symptoms, non-compliance or decreased feeding, cost-effectiveness of the intervention (reported in narrative text) and the psychosocial health of the infant (different scales for psychomotor development, cognitive development, attention, memory, language).

Studies were excluded if they did not report any of the outlined outcomes.

### 2.2. Literature Search

The literature review was conducted on the following databases: Cochrane Controlled Trials Register (CENTRAL), MEDLINE, EMBASE, CINAHL, PsycINFO, ERIC, Sociofiles, HMIS (Health Management Information Consortium), CAB Global Health (https://www.cabi.org/publishing-products/online-information-resources/global-health/), the WHO nutrition databases (http://www.who.int/nutrition/databases/en/), Popline (https://www.popline.org), Epistemonikos (https://www.epistemonikos.org/en/), Social Science Citation Index, Dissertation Abstracts International, and WHO Global Health Index which covers the WHO Regional journals from Latin America (LILACS), Africa (AFRO) etc. We also searched the web sites of selected development agencies or research firms (for example, JOLIS, IDEAS, IFPRI, NBER, USAID, World Bank and Eldis.org). The trials registry Clinicaltrials.gov and WHO’s ICRTP were searched for ongoing trials. Additionally, EPOC filters were used for quantitative designs (EPOC 2017b).

Every effort was made to contact the relevant organization, institutions and experts for the identification of unpublished and ongoing studies. Google Scholar and the Web of Sciences were searched for citations and the references sections and annotated bibliographies were cross referenced for bonus eligible studies.

### 2.3. Data Collection and Analysis

**Figure 1:**
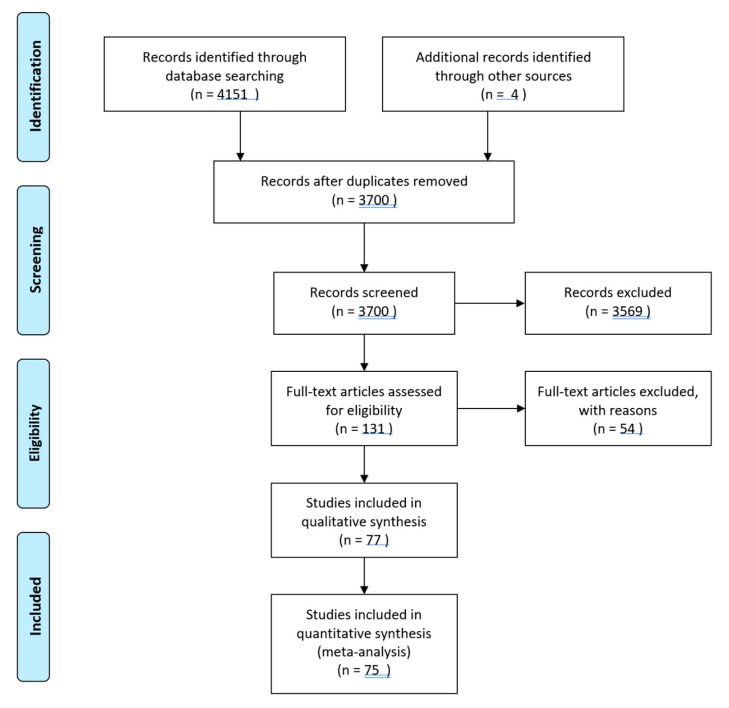
Search flow chart displaying the number of reports screened and then finally included in the review. *n* = number of studies.

Data collection and analysis was conducted in accordance with the Cochrane Handbook for Systematic Reviews of Interventions [17]. The selected literature was double screened at the title and abstract level by two independent review authors. The resulting full-texts were similarly double-screened by two independent authors with reasons recorded for exclusions. Disagreements were resolved by the consultation of a third senior review author. Duplicates were excluded. Multiple reports of the same study were collated to ensure each study was the unit of interest in this review. The selection process was recorded sufficient to construct a Preferred Reporting Items for Systematic Reviews and Meta-Analyses (PRISMA) flow diagram (see Figure 1) [18].

Two independent review authors extracted data on data extraction sheets on Microsoft Excel. Independent data extraction sheets were matched, and disagreements were resolved by discussion and third senior review author was consulted, if the need arose. We used a piloted data collection form for study characteristics and outcome data. If any information from the study was unclear or missing or could not be from the publications, the authors were contacted for further clarification.

Data was exported and analyzed on RevMan v5.3. Risk ratios (RR) were used for dichotomous outcomes, mean differences (MD) were calculated for continuous outcomes and standard mean differences were calculated for continuous outcomes reporting the same outcome but measured on a different scale within the same meta-analysis. A 95% confidence interval was used for all estimates of effect. We analyzed outcomes from studies with multiple groups in an appropriate way to avoid double counting of participants by adding them as an appropriately duplicated entry with an added footnote specifying the differences between groups.

#### 2.3.1. Assessment of Risk of Bias in Included Studies

Two review authors independently assessed the risk of bias of randomized controlled trials using the Cochrane Collaboration Risk of Bias tool [17] for selection bias, performance bias, attrition bias, reporting bias and other sources of bias. The risk of bias was rated either “high”, “low” or “unclear”.

For non-randomized controlled trials, controlled before-after studies and interrupted time series, we used EPOC methods (EPOC 2017a) to assess the risk of bias according to the following domains: random sequence generation, allocation concealment, baseline outcome measurements, baseline characteristic, incomplete outcomes, knowledge of allocated interventions adequately prevented during the study, protection against contamination, selective outcome reporting and other sources of bias. The risk of bias was rated either “high”, “low” or “unclear”.

#### 2.3.2. Assessment of Quality Evidence

A “Summary of Findings” table was constructed for all primary outcomes using the Grading of Recommendations Assessment, Development and Evaluation (GRADE) criteria [19] which involves consideration of within-study risk of bias (methodological quality), directness of evidence, heterogeneity, precision of effect estimates and risk of publication bias. The tool then rated quality of evidence as “high”, “moderate”, “low” and “very low”. Observational studies stood to be upgraded if they exhibited a large magnitude of effect, dose-response relationships and/or effect of plausible residual confounding as they begin at a “low” level of quality of evidence.

Breastfeeding education interventions had the following outcomes GRADEd: rates of early initiation of breastfeeding (within an hour of birth), rates of exclusive breastfeeding (at 3 and 6 months of age) and rates of continued breastfeeding (at 12, 18 and 24 months)

Complementary feeding education and provision studies and supplementary feeding interventions had the following outcomes GRADEd: mean Z-score for height-for-age, mean Z score for weight-for-height, mean Z score for weight-for-age, stunting, wasting, underweight.

#### 2.3.3. Subgroup Analysis, Investigation of Heterogeneity and Sensitivity Analysis

We conducted the following subgroup analyses where there were at least three studies in each sub-group, in adherence with Cochrane collaboration recommendations. Subgroups were divided by setting (home-based, facility-based or community-based), timing of intervention (prenatal, postnatal or a combination), duration of intervention (less than or equal to six months or greater than six months), observer (self-reported or clinically observed outcomes), moderators (community health worker (CHW)/volunteers or healthcare professionals) and by training of personnel (with WHO or United Nations International Children’s Education Fund (UNICEF) materials or from other sources) for breastfeeding education studies. Complementary feeding and supplementary feeding studies were subgroup analyzed by the level of food security of their settings using information available in the full texts of the articles. Sensitivity analyses were also performed to consider the impact of the following: high risk of bias in allocation concealment methodology, high risk of attrition bias due to lost participants and differences in outcomes due to different methods of calculating cluster adjustment

## 3. Results

### 3.1. Study Selection

We identified 4151 records from the search of multiple databases. After title and abstract screening and removal of duplicates, 131 studies were selected for full text screening. After full-text screening, 69 studies were excluded, and 77 studies were accepted for inclusion in the review. Four articles were also added from cross referencing of included studies. Of the 77 studies [20,21,22,23,24,25,26,27,28,29,30,31,32,33,34,35,36,37,38,39,40,41,42,43,44,45,46,47,48,49,50,51,52,53,54,55,56,57,58,59,60,61,62,63,64,65,66,67,68,69,70,71,72,73,74,75,76,77,78,79,80,81,82,83,84,85,86,87,88,89,90,91,92,93,94,95,96,97], 38 were on breastfeeding education interventions [21,22,23,24,25,26,27,29,32,33,37,38,39,42,44,49,51,53,54,56,57,58,63,65,66,67,68,70,73,75,81,83,87,88,91,93,94], 28 were on complementary feeding [20,28,30,31,34,43,46,48,55,60,61,62,64,69,71,72,74,76,77,78,79,80,82,85,92,93,95,96] and 12 investigated supplementary feeding interventions. The last search was carried out on 15 April 2019.

The included studies consisted of 44 individually randomized trials [20,21,22,23,24,25,29,30,32,33,35,39,40,42,45,48,51,54,56,58,59,62,63,64,66,69,70,71,72,75,76,77,79,81,85,86,87,88,89,91,92,93,95,96], 22 cluster randomized trials [26,27,28,31,34,37,38,41,43,44,46,47,49,50,53,55,57,65,67,73,74,80,82,83,84] and five quasi-experimental studies [52,61,68,90,94]. One study consisted of two parts, a longitudinal study and a randomized controlled trial [60]. One study was a non-randomized controlled trial [78]. (see Tables S1–S9 in Supplementary Data)

Seventy-one studies published between 1980 and 2019 involving 739, 343 participants met the inclusion criteria for this review (see Table S6: Characteristics of included studies in Supplementary Data 1). Outcome analysis data were contributed by 75 studies [20,21,22,23,24,25,26,27,28,29,30,31,32,33,34,35,37,38,39,40,41,42,43,44,45,46,47,48,49,50,51,53,54,55,56,57,58,59,60,61,62,63,64,65,66,67,68,69,70,71,72,73,74,75,76,77,78,79,80,81,82,83,84,85,86,87,88,89,90,91,92,93,94,95,96]. There were 26 studies [21,22,24,25,26,32,33,34,35,39,42,51,54,58,63,72,75,76,79,81,84,86,87,88,93,95] which were conducted in health care settings, whereas 51 [20,23,27,28,29,30,31,36,37,38,40,41,43,44,45,46,47,48,49,50,52,53,55,56,57,59,60,61,62,64,65,66,67,68,69,70,71,73,74,77,78,80,82,83,85,89,90,91,92,94,96] were conducted in home or community settings.

### 3.2. Comparison 1: Breastfeeding Education Interventions vs. Control

We included a total of 38 studies [21,22,23,24,25,26,27,29,32,33,37,38,39,42,44,49,51,53,54,56,57,58,63,65,66,67,68,70,73,75,81,83,86,87,88,91,94,95] as a part of the breastfeeding education comparison (see Table 1).

The breastfeeding education studies were con4ducted in a wide range of countries spanning four continents, i.e., twelve studies in Africa; one in Egypt [22]; two in Ghana [23,57]; two in Uganda [37,63]; one in Nigeria [38]; two in Tanzania [44,73]; two in Kenya [56,70]; one in South Africa [49]; one study in three countries of Africa [92]; Burkina Faso, Uganda and South Africa. Twenty-four studies were conducted in Asia; six in Bangladesh [26,27,51,53,89,95]; three in India [29,65,67]; one in Nepal [32]; two in Iran [39,75]; one in China [42]; one in Jordan [54]; one in Thailand [58]; one in Pakistan [83]; one in Malaysia [87]; two in Turkey [24,33]; one in Philippines [21]. One study was conducted in North America; in Mexico [66] and five in South America; in Brazil [25,68,81,86,93].

**Figure 2:**
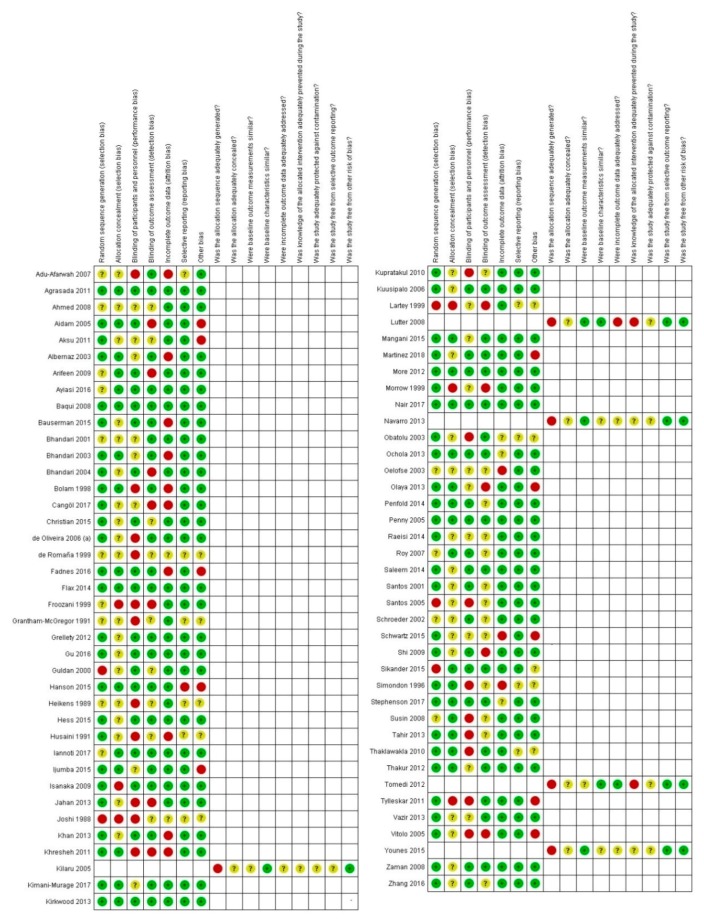
Traffic light plots presenting the risk of bias found in studies. Red circles represent a high risk of bias, green dots represent a low risk of bias and yellow dots represent an unclear risk of bias.

The minimum population size was 60 infant mother pairs in Ahmed et al. [22] and the maximum population size was 113,816 females in Baqui et al. [27]. The oldest average age among participants was 30.75 months in Younes et al. [94]. The timing of the intervention was prenatal in four studies; postnatal in 17 studies; and both prenatal and postnatal in 17 studies. In 21 studies, the intervention was provided by community health workers. In 10 studies, intervention was provided by health care professionals and 8 studies had study investigators as the providers respectively. Twelve studies’ training was sourced from WHO materials. Thirteen studies detailed training which was not based on WHO authored resources. The duration of training varied greatly from a minimum of two days in Arifeen et al. [26] and Sikander et al. [84] to a maximum of nine to 14 days in Nair et al. [67]. The training details are expanded in Table S6 (see Supplementary Data S1). The training methods were not specified in 13 studies, thus, they could not be included in the training/timing of training subgroup analyses. The risk of bias by study is enumerated in Figure 2.

Breastfeeding intervention may have caused a 20% increase in the prevalence of early initiation of breastfeeding, as reported by 14 studies [24,25,27,33,38,44,49,51,57,63,73,75,88]. Sensitivity analysis for high risk allocation concealment showed a 20% increase (RR 1.20, 95% CI 1.11 to 1.29; 10 studies; 83,565 participants) as ten studies remained [25,27,38,44,49,57,63,73,88]. Sensitivity analysis for attrition bias caused a 20% increase (RR 1.20, 95% CI 1.11 to 1.29; 12 studies; 83,868 participants) due the removal of two studies [25,33]. Subgroup analysis by setting showed facility-based [24,25,27,33,51,63,75,88] and community-based [38,49,57,73,83] had significant effects without significant heterogeneity between subgroups. Subgroup analysis by timing of intervention showed postnatal [24,25,88] and prenatal/postnatal interventions [27,33,44,49,57,73,75,83] both had a significant effect, whereas prenatal interventions [38,51,63] had no effect on early initiation of breastfeeding. Subgroup analysis by duration of intervention showed interventions lasting less than six months [24,25,33,49,63,75,88] had a significant effect, whereas interventions lasting more than six months [27,38,44,51,57,73,83] had no effect. Subgroup analysis by moderators showed CHW/volunteers [24,27,38,44,49,57,63,73,83] and healthcare professionals [25,33,51,75,88] as moderators both had significant effects. Subgroup analysis by the content of training moderators received showed training from other resources [27,44,51,57,63,73,83] had a significant effect while WHO/UNICEF resources [24,25,49] training did not.

We are unsure of the 102% increase reported in rates of EBF at three months, due to the very low-quality of evidence found by GRADE analysis [22,29,39,42,66,91]. Sensitivity analysis for allocation concealment removed five studies [22,39,42,66,91] and had a 65% increase (RR 1.65, 95% CI 1.48 to 1.84; one study; 895 participants) on EBF at three months. Similarly, sensitivity analysis by attrition bias removed one study [29] and showed a 123% increase in EBF at three months. Subgroup analysis by setting showed interventions conducted in facilities [22,39,42] and community settings [29,66,92] both had significant effects. Subgroup analysis by duration of intervention showed intervention lasting less than six months and those lasting more than six months both had a significant effect on the rate of EBF at three months.

**Figure 3:**
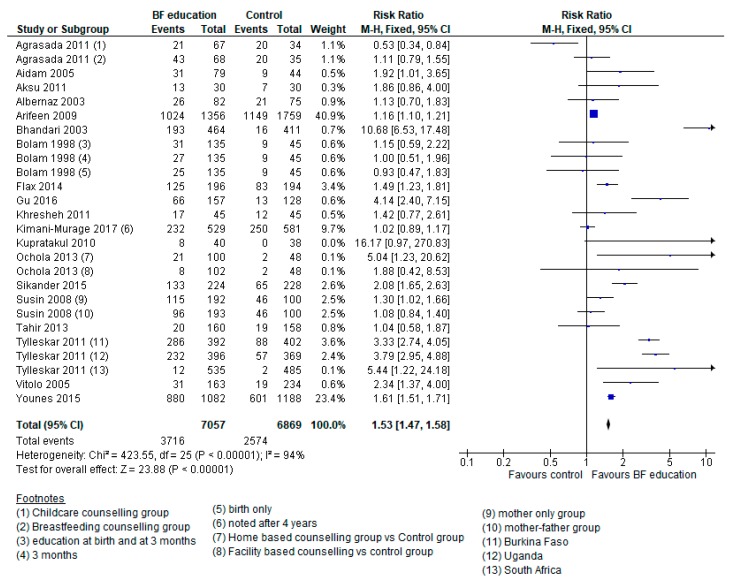
Forest plot displaying the meta-analysis of studies reporting on the effect of breastfeeding education interventions of the rate of exclusive breastfeeding at six months.

We are unsure of the 53% increase in breastfeeding interventions had on the rates of EBF at six months due to the very low-quality of evidence (see Figure 3) [21,23,24,25,26,29,32,38,42,54,56,58,70,83,86,87,91,93,94]. Sensitivity analysis for high risk allocation concealment removed five studies [24,42,58,92,94] and had a 37% increase (RR 1.37 95% CI 1.32 to 1.42; 14 studies; 10,527 participants; Heterogeneity: Chi^2^ *p* < 0.00001; I^2^ = 91%) rates of breastfeeding at six months. Similarly, sensitivity analysis for attrition bias removed five studies [25,29,32,54,70] and had a 47% increase (RR 1.47, 95% CI 1.42 to 1.52; 14 studies; 11966 participants) in EBF at six months. Subgroup analysis for setting showed facility [21,24,25,26,32,42,54,58,86,87,93] and community-based [23,29,38,56,70,83,91,94] interventions both had significant effects. Subgroup analysis by timing of intervention showed postnatal [21,24,25,26,32,42,54,56,86,87,93,94] and prenatal/postnatal interventions [23,58,70,83,91] both had significant effects.

Breastfeeding interventions had no effect on HAZ, as reported by six studies [26,37,53,67,68,81]. When filtering studies for risk of biased allocation concealment, three studies remained [26,37,67], which suggested a 0.17 standard deviation increase (MD 0.17, 95% CI 0.04 to 0.30; three studies; 3380 participants). Similarly, sensitivity analysis for attrition bias left two studies [26,67], indicating a 0.21 standard deviation increase (MD 0.21. 95% CI 0.10 to 0.31, two studies, 2917 participants). Subgroup analysis by timing of intervention indicated both purely postnatal [26,37,81] and prenatal/postnatal [53,67,68] had no effect.

Breastfeeding interventions had no effect on WAZ [37,53,67], WHZ [26,53,67], prevalence of stunting [26,27,37,67,68,81], wasting [26,67], underweight [27,37,67], neonatal mortality [27,65], nor on infant mortality [26,67]. Sensitivity analyses by high risk allocation concealment and attrition bias had no effect on the results of WAZ, stunting, wasting, infant mortality.

Breastfeeding education had a 24% decrease on the rates of diarrhea as reported by eight studies [21,26,29,39,66,68,89,95]. Sensitivity analysis for high risk of selection bias removed four studies [39,66,68,94] and indicated a 24% decrease (RR 0.76, 95% CI 0.67 to 0.85; four studies; 1613 participants). Sensitivity analysis by attrition bias removed two studies and indicated a 29% decrease (RR 0.71, 95% CI 0.63 to 0.81; six studies; 1863 participants) in diarrheal diseases. Subgroup analysis by setting showed community-based interventions [29,66,68,94] and facility-based interventions [21,26,39,89] both prevented diarrheal disease by similar magnitudes. Subgroup analysis by duration of intervention showed interventions lasting less than six months [21,66,88] and those lasting more than six months [26,29,39,68,94] both had significant effects on diarrheal disease.

Breastfeeding education had no effect on the incidence of infection, as reported by three studies [26,54,63]. Sensitivity analysis by allocation concealment methodology was not possible as all studies were at a low risk of bias. However, sensitivity analysis for attrition bias removed one study [54] and still showed no effect on the incidence of infections.

### 3.3. Comparison 2a: Complementary Feeding Education

We included 12 studies in the complementary feeding education comparisons [30,31,43,55,74,76,77,79,82,92,93,95].

Five studies reported data on WAZ [43,74,76,79,95], HAZ [43,74,76,79,95] and WHZ [74,76,79,95], six reported prevalence of stunting [30,31,43,74,77,92], three on the prevalence of wasting [30,43,77] and four studies reported data on weight [30,31,79,82] and height gain [30,31,79,82].

The complementary feeding education interventions spanned two continents. Asia housed nine studies; three in India [30,31,92], two in China [43,82], two in Pakistan [77,95] and two in Bangladesh [55,76]. Three studies were carried out in South America; two studies were carried out in Brazil [79,93] and one in Peru [74].

The range of the populations was from 212 at its minimum [76] and 1025 at its maximum [31]. The ages of participants ranged from 37 weeks of gestation at the minimum to 15.45 months at the maximum. There was also some variation in the intricacies of the interventions provided. The risk of bias by study is enumerated in Figure 2.

Complementary feeding education interventions caused a 0.41 SD increase in WAZ in food secure settings [43,74,79,95] and a 0.47 SD increase in food insecure settings [76] when compared with control groups (see Table 1).

Complementary feeding interventions had a significant effect in HAZ in food secure settings [43,74,79,95] and caused a 0.26 SD increase in food insecure settings [76] (see Table 1).

Complementary feeding education interventions had no significant effect on WHZ in food secure settings [74,79,95] and a 0.50 SD increase in a food insecure setting [76] when compared with control groups (see Table 1).

Complementary feeding education interventions caused a non-significant 50% decrease in the prevalence of stunting in food secure settings [43,74,77] and a 35% decrease in food insecure settings [30,31,92] when compared with control groups (see Table 1).

### 3.4. Comparison 2b: Complementary Feeding Provision with or without Education vs. Control

Seventeen trials were included that addressed the effect of complementary feed provision studies regardless of the provision of education. HAZ was reported by 12 studies, WAZ was reported by 10 studies, WHZ was reported by 10 studies, stunting was reported by seven studies, wasting was reported by five studies, weight was reported by four studies and height gain by three studies (see Table 1).

The studies spanned three continents. Seven studies were conducted in Africa i.e., two in Ghana [20,60], two in Malawi [62,85], and one each in Burkina Faso [46], Democratic Republic of Congo [28], Nigeria [69] and South Africa [71]. Five studies were conducted in South America i.e., two in Ecuador [48,61] and one each in Guatemala [64], Colombia [72] and Brazil [78]. Four studies were conducted in Asia i.e., one each in India [30], Bangladesh [34], China [96] and Vietnam [80].

Complementary feeding provision had a significant effect on HAZ in food insecure settings [20,28,34,46,48,61,62,69,71,72,80,85] but had no effect in a food secure setting [78] when compared with control groups (see Table 1).

Complementary feeding provision had significant effects on the prevalence of stunting in food secure [97] and food insecure settings [28,34,46,48,61,62,80] when compared with control groups (see Table 1).

### 3.5. Comparison 3: Supplementary Feeding Programs vs. Control

The studies spanned five continents. Africa housed five studies i.e., two in Niger [41,50], two in Malawi [59,89], one in Kenya [90]. Two studies were conducted in Asia i.e., one in Indonesia [47] and one in Vietnam [80]. Two studies were conducted in only North America in Jamaica [40,45]. One study was conducted in only South America in Brazil [35] and Peru [36]. Simondon et al. [84] was conducted in four different countries in Congo, Senegal, Bolivia and New Caledonia (i.e., Africa, Africa, South America and Oceania, respectively).

The risk of bias by study is enumerated in Figure 2. Only 11 of the 13 supplementary feeding F studies contributed to the meta-analysis [35,40,41,45,47,50,59,80,84,89,90].

There was no composite effect of supplementary feeding interventions on HAZ. Supplementary feeding interventions had a significant effect on HAZ in a food secure setting [47] but had no significant effect in food insecure settings [50,59,80,89,90] when compared to control groups (see Table 1).

Supplementary feeding interventions had a significant effect (MD 0.30; 95% CI 0.10 to 0.50; one study; 113 participants) on WAZ in a food secure setting [47] but had no significant effect (MD 0.19; 95% CI −0.18 to 0.55; four studies; 598 participants) in food insecure settings [59,80,89,90] (see Table 1).

Supplementary feeding interventions had a significant composite effect (MD 0.15; 95% CI 0.08 to 0.22; six studies; 3664 participants; GRADE) on WHZ when compared with control groups. All included studies were from food insecure settings [40,50,59,80,89,90] (see Table 1).

Supplementary feeding interventions had no significant composite effect (RR 1.31; 95% CI 0.95 to 1.81; four studies; 1512 participants) on the prevalence of stunting. Supplementary feeding similarly had no effect in food secure (RR 1.29; 95% CI 0.88 to 1.89; three study; 1335 participants) [41,80,91] and food insecure (RR 1.81; 95% CI 0.34 to 9.62; one study; 177 participants) settings [35] (see Table 1).

Supplementary feeding interventions had no significant composite effect (RR 0.80; 95% CI 0.55 to 1.17; four studies; 4299 participants) on the prevalence of wasting. All included studies were in food insecure settings [41,50,80,90] (see Table 1).

Supplementary feeding interventions caused a significant decrease (RR 0.61; 95% CI 0.38 to 0.97; two studies; 4757 participants) in infant mortality rates when compared with control groups. All included studies were conducted in food insecure settings [41,50] (see Table 1).

Supplementary feeding interventions had no significant composite effect (MD 0.06; 95% CI −0.01 to 0.12; five studies; 845 participants; GRADE) on weight gain when compared with control groups. Similarly, no effect was seen in a food secure setting (MD −0.07; 95% CI −0.15 to 0.01; one study; 90 participants). However, food insecure settings saw a statistically significant 0.08 kg increase (MD 0.08; 95% CI 0.01 to 0.14; five studies; 755 participants) in mean weight gain (see Table 1).

Supplementary feeding intervention had no significant composite effect (MD 0.13; 95% CI −0.03 to 0.29; five studies; 832 participants) on mean height gain when compared with control groups. Similarly, no effect was detected in food secure nor food insecure settings (see Table 1).

## 4. Discussion

This review includes 66 studies of which 38 studies contributed to breastfeeding education comparisons, 17 studies contributed to complementary feed provision regardless of, 12 studies contributed to complementary feeding education only and 13 studies contributed to supplementary feeding comparisons. All primary outcomes were reported except for continued breastfeeding at 12 and 24 months of age. The included studies span 29 countries from four continents, all contributing to a comprehensive review of evidence.

Breastfeeding intervention caused a 20% increase in rates of early initiation of breastfeeding, a 102% increase in the rates of EBF at 3 months and 53% increase in EBF at six months. It was also associated in a 24% decrease in diarrheal disease. Complementary feeding was associated in 0.12 SD increase in height-for-age Z-scores, a 13% decrease in stunting and 11% decrease in wasting. Supplementary feeding caused a 39% decrease in the rates of infant mortality. Other outcomes did not report significant associations.

Subgroup analyses revealed information on the study characteristics that went into producing significant results. Early initiation of breastfeeding was only supported by significant effects in facility- and community-based interventions, postnatal or prenatal/postnatal CHW/volunteer or healthcare professional led, interventions lasting more than six months, trained on other resources. Moreover, strictly prenatal interventions and those lasting less than six months had no effect. That is to say that the moderators’ profession and setting do not necessarily matter, but that the duration and timing of intervention are more crucial points in study design aiming to maximize early initiation of breastfeeding. It is worth mentioning that healthcare professional moderated interventions had double the effectiveness of those moderated by CHW/volunteers.

Exclusive breastfeeding at 3 months of age was supported by significant effects in both community and facility-based interventions. Of note, facility-based interventions were more than 3 times more effective than the community-based interventions.

Exclusive breastfeeding at 6 months of age was supported by significant effects in all subgroups allowing the conclusion that the outlined subgroups can all be utilized for significant improvements in breastfeeding practices at 3 months. However, it was found that community-based interventions delivered in prenatal and postnatal period for periods less than six months based on WHO/UNICEF materials had more efficacious magnitudes of effect than their subgroup counterparts. This information can be used to design a maximally effective intervention if the goal is maximizing EBF at 3 months.

Complementary feeding education had significant effects on WAZ and HAZ in all food security settings. Complementary feeding education had significant effects on WHZ in only food insecure settings.

Complementary feed provision interventions had significant effects on HAZ in food insecure settings and on stunting in food secure and insecure settings. Supplementary feeding interventions had significant effects on HAZ and WAZ in food secure settings and on WHZ and infant mortality in food insecure settings.

The quality of outcomes was determined with the GRADE method [35]. In breastfeeding education interventions, early initiation of breastfeeding was graded low due to very serious inconsistencies, EBF at 3 months was graded very low due to serious inconsistency and EBF at 6 months was graded very low due to serious risk of bias and very serious inconsistency. Every effort was made to minimize the sources of bias including independent screening and assessment of data.

Our findings appear to agree with other systematic reviews of the relevant literature. Sinha et al. [97] reported a 66% increase in early initiation of breastfeeding in studies conducted in LMICs. The same study also conducted subgroup analyses and found facility-based interventions caused a 12% increase in early initiation of breastfeeding, which compare closely with the 18% increase observed in our subgroup analyses. Sinha et al. [98] also found community-based interventions caused an 86% increase in early initiation of breastfeeding which is significant but much larger than the 17% increase found in our subgroup analyses. It should be noted that the subgroup analyses do not strictly capture the effect of LMICs.

Rollins et al. [1,98] also had a significant 11% increase in rates of early breastfeeding which was in agreement with our findings but to a smaller magnitude.

Balogun et al. [99] was also in agreement, finding a 22% increase in the rates of early breastfeeding. Additionally, subgroup analyses performed by moderators showed healthcare professional led interventions had a 43% increase in early initiation of breastfeeding. These results were significant and in agreement with our subgroup analyses finding a 33% increase in healthcare professional moderated interventions’ effect on early initiation of breastfeeding.

Rates of EBF at six months were reported in two systematic reviews [100,101]. Sinha’s review found interventions in LMICs increased rates of EBF at six months by 69% [101]. This compares closely as a significant effect with the 53% increase found in our meta-analysis. Additionally, Sinha et al. conducted a subgroup analysis by setting which found facility-based interventions to have a significant 46% increase in EBF at six months [101]. This is again concordant with the 20% increase concluded by our subgroup analysis.

Kim et al. included an extensive review of subgroups very similar to ours, allowing for a rich parallel comparison [97]. As a composite outcome, they found a 173% increase in the odds of EBF at six months (compared to the 53% increase found in our meta-analysis) [97]. Subgroup analysis by setting showed community-based interventions had a 177% increase in the odds of EBF at six months (compared to the 90% increase found in our meta-analysis) [97]. Kim et al. also conducted a subgroup analysis by timing of interventions and found prenatal interventions had no effect on EBF at six months, postnatal interventions caused 165% increased odds and pre/postnatal interventions caused an 83% increase [97]. Again, this is in agreement with our findings of prenatal interventions having no effect, postnatal interventions causing an 89% increase and pre/postnatal interventions causing a 71% increase in the rates of EBF at six months. Overall, wherever possible to draw a comparison, Kim et al.’s findings seems to be qualitatively in agreement with our findings [97]. However, Kim et al.’s findings consist of larger magnitudes of effect and are calculated as odd ratios [97].

Complementary feeding studies were divided into those providing only complementary feeding education and those providing only complementary feeding provision. Arikpo et al. [102] were studying only education interventions and reported no effects of interventions on the prevalence of stunting, wasting and underweight. Similarly, our review found no effect of complementary feeding education on stunting and wasting.

Lassi et al. [103] found significant effect of CF on HAZ and WAZ in food secure and food insecure settings which matched the findings of our review. Lassi et al. also found significant reductions in stunting in a food insecure setting, whereas our review included more studies but found no association in both food insecure and food secure settings.

Supplementary feeding programs were extensively analyzed as subgroups in Kristjansson et al.’s review [104]. This allowed for some comparison with RCT only outcomes of Kristjansson et al. [104] since the composite results included a wide variety of study designs. Subgroup analysis by LMICs showed there was no effect on WHZ and increases in weight gain (MD 0.12 kg), height gain (0.27 cm), change in WAZ (MD 0.15) and height gain (MD 0.85). These results are in complete agreement with the results of Kuusipalo et al. [59] as reviewed by us, as these outcomes were only reported by this study. Other supplementary feeding studies in the literature were not comparable, as, for example, Beaton et al. [105] focused on the effects of interval growth, Pollitt et al. [106] focused on the effect on cognitive performance.

The updated and extensive nature of this review offers much utility to policy-making bodies in the interest of maximizing resources for the betterment of infant nutrition. The present review offers such utility in breastfeeding education and practice as well as for the provision of complementary nutrition allowing for maximized growth and minimized states of malnutrition and disease. However, owing to the exclusive inclusion of studies from LMICs, the data presented is not necessarily generalizable outside of such socioeconomic settings.

## 5. Conclusions

We believe the present review provides a comprehensive review of the literature and has much to contribute to the literature in the forms of extensive subgroup analyses. The updated and extensive nature of this review offers much utility to policy-making bodies in the interest of maximizing resources for the betterment of infant nutrition in LMICs. The present review offers much utility in breastfeeding education and practice as well as for the provision of complementary nutrition allowing for maximized growth and minimized states of malnutrition and disease in the socioeconomic settings of our interest. The beneficial effects of breastfeeding interventions on future research in the area of supplementary feeding would allow more robust meta-analyses as current data is largely inconclusive. Another point of research could be investigating the cost effectiveness of such interventions as has been alluded to by many of the included studies but only reported by two of the studies.

## Figures and Tables

**Table 1:** Estimates of effect of the interventions and outcomes.

Outcomes	MD	RR	95% CI	*n*	No. of Studies	GRADE	*p*-Value
***Breastfeeding Education Interventions***							
**Early initiation of breastfeeding**		**1.20**	**1.12 to 1.28**	**84092**	**14**	**LOW**	**<0.001**
**EBF @ 3 months**		**2.02**	**1.88 to 2.17**	**4063**	**6**	**VERY-LOW**	**<0.001**
**EBF @ 6 months**		**1.53**	**1.47 to 1.58**	**13926**	**19**	**VERY-LOW**	**<0.001**
HAZ	0.10		−0.04 to 0.25	5620	6	-	0.17
WAZ	−0.04		−0.12 to 0.05	4565	3	-	0.42
WHZ	0.01		−0.07 to 0.09	4514	3	-	0.83
stunting		1.00	0.88 to 1.14	6518	6	-	0.99
Underweight		1.31	0.79 to 2.16	3448	3	-	0.30
WAWasting		0.94	0.86 to 1.03	3925	2	-	0.19
Neonatal mortality (%)		1.10	0.90 to 1.24	22752	2	-	0.35
Infant mortality (%)		0.86	0.73 to 1.02	35943	2	-	0.08
**Diarrheal disease**		**0.76**	**0.67 to 0.85**	**4585**	**8**	**-**	**<0.001**
Incidence of infection		1.96	0.65 to 5.93	1831	3	-	0.23
***Complementary Feeding Education—Food Secure Settings***							
**WAZ**	**0.41**		**0.07 to 0.75**	**1562**	**F4**	**HIGH**	**0.02**
**HAZ**	**0.29**		**0.04 to 0.54**	**1560**	**4**	**MODERATE**	**0.03**
WHZ	0.22		−0.03 to 0.47	1065	3	MODERATE	0.08
Stunting		0.50	0.18 to 1.40	1006	3	LOW	0.19
Wasting		0.19	0.03 to 1.18	665	2	MODERATE	0.07
Weight gain (kg)	0.12		−0.12 to 0.37	894	2	MODERATE	0.33
Height gain (cm)	0.33		−0.46 to 1.12	894	2	LOW	0.42
***Complementary Feeding Education—Food Insecure Settings***							
**WAZ**	**0.47**		**0.35 to 0.59**	**572**	**1**	**LOW**	**<0.001**
**HAZ**	**0.25**		**0.09 to 0.41**	**572**	**1**	**HIGH**	**0.002**
**WHZ**	**0.50**		**0.35 to 0.65**	**572**	**1**	**HIGH**	**<0.001**
Stunting		0.65	0.42 to 1.01	1476	3	LOW	0.05
Wasting		1.05	0.15 to 7.26	178	1	MODERATE	0.07
Weight gain (kg)	0.01		−0.07 to 0.08	1213	2	LOW	0.33
Height gain (cm)	−0.09		−0.30 to 0.12	1214	2	LOW	0.42
***Complementary Feed Provision Interventions—Food Secure Settings***							
WAZ	−0.09		−0.34 to 0.16	172	1	LOW	0.48
HAZ	−0.12		−0.46 to 0.22	172	1	LOW	0.49
WHZ	−0.03		−0.28 to 0.22	172	1	LOW	0.81
**Stunting**		**0.47**	**0.37 to 0.59**	**2896**	**1**	**HIGH**	**<0.001**
Weight gain (kg)	−0.01		−0.24 to 0.22	172	1	LOW	0.93
Height gain (cm)	−0.23		−1.11 to 0.65	172	1	LOW	0.61
***Complementary Feed Provision Interventions—Food Insecure Settings***							
WAZ	0.34		−0.35 to 1.03	3570	10	VERY-LOW	0.33
**HAZ**	**0.14**		**0.04 to 0.24**	**8996**	**12**	**LOW**	**0.005**
WHZ	0.01		−0.01 to 0.03	8197	10	LOW	0.27
**Stunting**		**0.64**	**0.44 to 0.92**	**7894**	**7**	**LOW**	**0.02**
Wasting		0.87	0.74 to 1.01	7081	6	MODERATE	0.07
Weight gain (kg)	0.62		−0.02 to 1.26	986	3	VERY-LOW	0.06
Height gain (cm)	0.17		−0.11 to 0.44	924	3	MODERATE	0.24
***Supplementary Feeding Interventions***							
HAZ	0.11		−0.03 to 0.24	3724	6	LOW	0.13
WAZ	0.20		−0.12 to 0.52	711	5	VERY-LOW	0.23
**WHZ**	**0.15**		**0.08 to 0.22**	**3664**	**6**	**MODERATE**	**<10^−5^**
Stunting		1.31	0.95 to 1.81	1512	4	LOW	0.10
Wasting		0.80	0.55 to 1.17	4299	4	LOW	0.25
**Infant mortality (%)**		**0.61**	**0.38 to 0.97**	**4757**	**2**	**HIGH**	**0.04**
Weight gain (kg)	0.06		−0.01 to 0.12	845	5	LOW	0.08
Height gain (cm)	0.13		−0.03 to 0.35	832	5	LOW	0.11

Significant effect considered at *p* < 0.05. MD = mean difference. RR = risk ratio. HAZ = height-for-age z-scores. WAZ = weight-for-age z-scores. WHZ = weight-for-height z-scores. HAZ < 2 = stunting. WAZ < 2 = wasting. WHZ < 2 = underweight. EBF: exclusive breastfeeding. significant estimates are in BOLD.

## Supplementary Materials

The following are available online at https://www.mdpi.com/2072-6643/12/3/722/s1. Table S1: Breastfeeding education meta-analyses; Table S2: Complementary feeding education and provision meta-analyses; Table S3: Supplementary feeding meta-analyses; Table S4: Single study outcomes; Table S5: Search strategy; Table S6: Characteristics of included studies; Table S7: Summary of findings table of breastfeeding education; Table S8: Summary of findings table of complementary feeding; Table S9: Summary of findings table of supplementary feeding.
