# Supplementary material for: Impact of Infant and Young Child Feeding (IYCF) Nutrition Interventions on Breastfeeding Practices, Growth and Mortality in Low- and Middle-Income Countries: Systematic Review
Source: Nutrients. 2020 Mar 10;12(3):722. doi: 10.3390/nu12030722 (PMC7146402; doi:10.3390/nu12030722)
Supplement: Supplementary file 1 [file nutrients-12-00722-s001.pdf]

Supplementary data S1

Table S1: Breastfeeding education meta-analyses

Outcome	Composite effect	Subgroup by Setting	Subgroup by timing of intervention	Subgroup by duration of intervention	Subgroup by moderators	Subgroup by training of supervisors
Early initiation of breastfeeding	RR 1.20; 95% CI 1.12 to 1.28, Tau² = 0.01; Chi² = 243.17, df = 14 (P < 0.00001); I² = 94%;<br>14 studies [1-14]<br>84092 participants	1. Facility-based: RR 1.18; 95% CI 1.03 to 1.36; Eight studies [1-4, 8, 10, 12, 14]; Tau² = 0.03; Chi² = 69.55, df = 7 (P < 0.00001); I² = 90%.<br>2. Community-based: RR 1.17; 95% CI 1.07 to 1.28; five studies [5, 7, 9, 11, 13]; Tau² = 0.01; Chi² = 13.16, df = 4 (P = 0.01); I² = 70%	1. Prenatal: RR 1.06; 95% CI 0.98 to 1.14; Three studies [5, 8, 10]; Chi² = 50.74, df = 2 (P < 0.00001); I² = 96%<br>2. Postnatal: RR 1.32; 95% CI 1.09 to 1.59; Three studies [5, 10, 15]; Chi² = 17.78, df = 2 (P = 0.0001); I² = 89%<br>3. Prenatal/Postnatal: RR 1.25; 95% CI 1.24 to 1.27; Eight studies [3, 4, 6, 7, 9, 11-13]; Chi² = 236.01, df = 8 (P < 0.00001); I² = 97%	1. ≤6 months: RR 1.10; 95% CI 0.96 to 1.25; seven studies [1, 2, 4, 7, 10, 12, 14]; Tau² = 0.02; Chi² = 29.47, df = 6 (P < 0.0001); I² = 80%<br>2. >6 months: RR 1.28; 95% CI 1.18 to 1.38; seven studies [3, 5, 6, 8, 9, 11, 13]; Tau² = 0.01; Chi² = 147.01, df = 7 (P < 0.00001); I² = 95%	1. CHW/volunteers: RR 1.16; 95% CI 1.07 to 1.25; nine studies [1, 3, 5-7, 9-11, 13]; Tau² = 0.01; Chi² = 233.63, df = 9 (P < 0.00001); I² = 96%<br>2. Healthcare professionals: RR 1.33; 95% CI 1.58; five studies [2, 4, 8, 12, 14]; Tau² = 0.02; Chi² = 13.22, df = 4 (P = 0.01); I² = 70%	1. WHO/UNICEF materials: RR 1.04; 95% CI 0.94 to 1.15; three studies [1, 2, 7]; Tau² = 0.00; Chi² = 2.51, df = 2 (P = 0.29); I² = 20%<br>2. Other sources: RR 1.20; 95% CI 1.08 to 1.26; seven studies [3, 6, 8-11, 13]; Tau² = 0.01; Chi² = 206.20, df = 7 (P < 0.00001); I² = 97%
Exclusive breastfeeding at 3 months of age	RR 2.02; 95% CI 1.88 to 2.17, Chi² = 35.63, df = 8 (P < 0.0001); I² = 78%<br>Six studies [16-21]<br>4063 participants	1. Facility-based: RR 4.30; 95% CI 1.97 to 6.21: three studies [16, 18, 19]; Chi² = 2.35, df = 2 (P = 0.31); I² = 15%<br>2. Community-based: RR 1.90; 95% CI 1.76 to 2.04, three studies [17, 20, 21]: Chi² = 14.67, df = 5 (P = 0.01); I² = 66%				
Exclusive breastfeeding at 6 months	RR 1.53; 95% 1.47 to 1.58, Chi² = 423.55, df = 25 (P < 0.00001); I² = 94%<br>19 studies [1, 2, 5, 13, 17, 19, 21-33]<br>13926 participants	1. Community-based: RR 1.20; 95% CI 1.15 to 1.25; 11 studies [1, 2, 19, 22, 24-26, 28, 30-32]; Chi² = 47.70, df = 14 (P < 0.0001); I² = 71%<br>2. Facility-based: RR 1.90; 95% CI 1.80 to 2.00; eight studies [5, 13, 17, 21, 23, 27, 29, 33]; Chi² = 228.35, df = 10 (P < 0.00001); I² = 96%	1. Postnatal: RR 1.29; 95% CI 1.24 to 1.33; 12 studies [1, 2, 19, 22, 24-27, 30-33]; Chi² = 123.38, df = 15 (P < 0.00001); I² = 88%<br>2. Prenatal and postnatal: RR 3.08; 95% CI 2.72 to 3.49; five studies [13, 21, 23, 28, 29]; Chi² = 18.98, df = 7 (P = 0.008); I² = 63%	1. ≤ 6 months: RR 1.77; 95% CI 1.63 to 1.91; 12 studies [1, 2, 19, 22, 24-27, 30-33]; Chi² = 114.43, df = 7 (P < 0.00001); I² = 94%<br>2. > 6 months: RR 1.50; 95% CI 1.45 to 1.56; five studies [13, 21, 23, 28, 29]; Chi² = 306.00, df = 17 (P < 0.00001); I² = 94%	1. CHW/volunteers: RR 1.64; 95% CI 1.49 to 1.80; six studies [1, 5, 13, 17, 25, 27]; Chi² = 114.43, df = 7 (P < 0.00001); I² = 94%<br>2. Healthcare professionals: RR 1.50; 95% CI 1.45 to 1.56; 13 studies [2, 19, 21-24, 26, 28-33]; Chi² = 306.00, df = 17 (P < 0.00001); I² = 94%	1. WHO resources: RR 2.24; 95% CI 2.04 to 2.45; eight studies [1, 2, 17, 21, 23, 27, 29, 31]; Chi² = 220.54, df = 10 (P < 0.00001); I² = 95%<br>2. Other resources: RR 1.33; 95% CI 1.28 to 1.38; five studies [13, 22, 24, 25, 33]; Chi² = 103.40, df = 6 (P < 0.00001); I² = 94%
Height-for-age Z-scores (HAZ)	MD 0.10; 95% CI -0.04 to 0.25, Tau² = 0.03; Chi² = 195.24, df = 5 (P < 0.00001); I² = 97%<br>Six studies [15, 24, 34-37]<br>5620 participants		1. Postnatal: MD 0.09; 95% CI -0.09 to 0.27; three studies [24, 34, 37]; Tau² = 0.01; Chi² = 4.19, df = 2 (P = 0.12); I² = 52%<br>2. Prenatal and postnatal: MD 0.13; 95% CI -0.12 to 0.39; three studies [15, 35, 36]; Tau² = 0.05; Chi² = 58.22, df = 2 (P < 0.00001); I² = 97%			
Weight-for-age Z-scores	MD -0.04; 95% CI -0.12 to 0.05; Tau² = 0.00; Chi² = 6.62, df = 2 (P = 0.04); I² = 70%<br>Three studies [15, 34, 35]<br>4565 participants					

Weight-for-height Z-scores (WHZ)	MD 0.01; 95% CI -0.07 to 0.09, Tau² = 0.00; Chi² = 24.23, df = 2 (P < 0.00001); I² = 92%<br>Three studies [15, 24, 35]<br>4514 participants					
Stunting	RR 1.00; 95% 0.88 to 1.14, Tau² = 0.01; Chi² = 17.86, df = 5 (P = 0.003); I² = 72%<br>Six studies [3, 24, 34-37]<br>6518 participants					
Underweight	RR 1.31; 95% CI 0.79 to 2.16; Tau² = 0.18; Chi² = 19.49, df = 2 (P < 0.0001); I² = 90%<br>Three studies [3, 34, 35]<br>3448 participants					
Wasting	RR 0.94; 95% CI 0.86 to 1.03; Tau² = 0.00; Chi² = 0.24, df = 1 (P = 0.62); I² = 0%<br>Two studies [24, 35]<br>3925 participants					
Neonatal mortality	RR 1.10; 95% CI 0.90 to 1.34; Chi² = 14.15, df = 2 (P = 0.0008); I² = 86%<br>Two studies [3, 38]<br>22752 participants					
Infant mortality	RR 0.86; 95% CI 0.73 to 1.02; Tau² = 0.00; Chi² = 0.34, df = 1 (P = 0.56); I² = 0%<br>Two studies [24, 35]<br>35943 participants					
Diarrheal disease	RR 0.76; 95% CI 0.67 to 0.85; Chi² = 14.02, df = 8 (P = 0.08); I² = 43%<br>Eight studies [14, 17, 18, 20, 22, 24, 33, 36]<br>4585 participants	1. Community-based: RR 0.83; 95% CI 0.72 to 0.94, four studies [17, 20, 33, 36], Chi² = 3.75, df = 3 (P = 0.29); I² = 20%<br>2. Facility-based: RR 0.56; 95% CI 0.44 to 0.72, four studies [14, 18, 22, 24], Chi² = 5.08, df = 4 (P = 0.28); I² = 21%		1. ≤6 months: RR 0.62; 95% CI 0.48 to 0.79; three studies [14, 20, 22]; Chi² = 2.10, df = 3 (P = 0.55); I² = 0%<br>2. > 6 months: RR 0.80; 95% CI 0.70 to 0.91; five studies [17, 18, 24, 33, 36]; Chi² = 8.68, df = 4 (P = 0.07); I² = 54%		
Incidence of infections	RR 1.96; 95% CI 0.65 to 5.93; Tau² = 0.84; Chi² = 32.55, df = 2 (P < 0.00001); I² = 94%<br>Three studies [10, 24, 26]<br>1831 participants					

Table S2: Complementary feeding education and provision meta-analyses

Outcomes	Composite effect	Subgroup by setting	Subgroup by duration of intervention	Subgroup by moderators	Subgroup by training and supervision

Weight-for-age Z-scores (WAZ)	MD 0.13; 95% -0.02 to 0.28; Chi² = 8.68, df = 4 (P = 0.07); I² = 54%; Tau² = 0.07; Chi² = 112.51, df = 15 (P < 0.00001); I² = 87%<br>13 studies [39-51]<br>4543 participants	1. Community-based: MD 0.14; 95% CI -0.02 to 0.31; 10 studies [39-44, 46-48, 50]; Tau² = 0.07; Chi² = 97.30, df = 12 (P < 0.00001); I² = 88%<br>2. Facility-based: MD 0.06; 95% CI -0.39 to 0.51; three studies [45, 49, 51];Tau² = 0.13; Chi² = 12.14, df = 2 (P = 0.002); I² = 84%	1. ≤ 6 months: MD 0.08; 95% CI -0.18 to 0.34; seven studies [41, 44, 45, 47, 48, 50, 51]; Tau² = 0.11; Chi² = 49.59, df = 7 (P < 0.00001); I² = 86%<br>2. > 6 months: MD 0.17; 95% CI -0.09 to 0.42; four studies [39, 40, 43, 46]; Tau² = 0.09; Chi² = 59.88, df = 5 (P < 0.00001); I² = 92%	1. CHW/volunteers: MD 0.00; 95% CI -0.20 to 0.20; five studies [39, 43, 45, 47, 50]; Tau² = 0.07; Chi² = 55.01, df = 7 (P < 0.00001); I² = 87%<br>2. Healthcare professionals: MD 0.28; 95% CI 0.06 to 0.50; eight studies [40-42, 44, 46, 48, 49, 51]; Tau² = 0.08; Chi² = 46.80, df = 7 (P < 0.00001); I² = 85%	
Height -for-age Z-scores (HAZ)	MD 0.12; 95% CI 0.05 to 0.19; Tau² = 0.01; Chi² = 42.10, df = 19 (P = 0.002); I² = 55%<br>14 studies [39-52]<br>9443 participants	1. Community-based: MD 0.13; 95% CI 0.05 to 0.20; 11 studies [39-44, 46-48, 50, 52]; Tau² = 0.01; Chi² = 40.73, df = 16 (P = 0.0006); I² = 61%<br>2. Facility-based: MD 0.04; 95% CI -0.10 to 0.19; three studies [45, 49, 51]; Tau² = 0.00; Chi² = 0.58, df = 2 (P = 0.75); I² = 0%	1. ≤ 6 months: MD 0.13; 95% CI 0.04 to 0.22: nine studies [41, 42, 44, 45, 47-51]; Tau² = 0.00; Chi² = 9.96, df = 9 (P = 0.35); I² = 10%<br>2. > 6 months: MD 0.12; 95% CI 0.03 to 0.22; five studies [39, 40, 43, 46, 52]; Tau² = 0.02; Chi² = 31.62, df = 9 (P = 0.0002); I² = 72%	1. CHW/volunteers: MD 0.10; 95% CI 0.03 to 0.16; eight studies [39, 43, 45-47, 50-52]; Tau² = 0.01; Chi² = 23.19, df = 13 (P = 0.04); I² = 44%<br>2. Healthcare professionals: MD 0.19; 95% CI -0.04 to 0.42; six studies [40-42, 44, 48, 49]; Tau² = 0.05; Chi² = 17.14, df = 5 (P = 0.004); I² = 71%	
Weight-for-height Z-scores (WHZ)	MD 0.02; 95% CI -0.01 to 0.04; Tau² = 0.00; Chi² = 8334.56, df = 16 (P < 0.00001); I² = 100%<br>12 studies [39, 41-49, 51, 52]<br>12376 participants	1. Community-based: MD 0.01; 95% CI -0.01 to 0.04; nine studies [39, 41-44, 46-48, 52]; Tau² = 0.00; Chi² = 8315.07, df = 13 (P < 0.00001); I² = 100%<br>2. Facility-based: MD 0.04; 95% CI -0.48 to 0.57; three studies [45, 49, 51]; Tau² = 0.19; Chi² = 16.45, df = 2 (P = 0.0003); I² = 88%	1. ≤ 6 months: MD 0.11; 95% -0.20 to 0.42; six studies; Tau² = 0.11; Chi² = 34.31, df = 5 (P < 0.00001); I² = 85%<br>2. > 6 months: MD 0.00; 95% CI -0.02 to 0.02; four studies; Tau² = 0.00; Chi² = 8267.72, df = 8 (P < 0.00001); I² = 100%	1. CHW/volunteers: MD 0.01; 95% CI -0.01 to 0.03; five studies [39, 43, 45, 47, 52]; Tau² = 0.00; Chi² = 8316.58, df = 9 (P < 0.00001); I² = 100%<br>2. Healthcare professionals: MD 0.14; 95% CI 0.01 to 0.26; seven studies [41, 42, 44, 46, 48, 49, 51]; Tau² = 0.01; Chi² = 9.89, df = 6 (P = 0.13); I² = 39%	
Change in HAZ	MD 0.04; 95% CI -0.04 to 0.12; Chi² = 1.00, df = 2 (P = 0.61); I² = 0%<br>Two studies [50, 53]<br>680 participants				

Stunting	RR 0.87; 95% CI 0.77 to 0.98; Tau² = 0.04; Chi² = 60.47, df = 17 (P < 0.00001); I² = 72%<br>12 studies [39-43, 46, 52, 54-58]<br>16002 participants	1. CHW/volunteers: RR 0.87; 95% CI 0.73 to 1.05; six studies [43, 46, 52, 55, 57, 58]; Tau² = 0.07; Chi² = 54.98, df = 11 (P < 0.00001); I² = 80%<br>2. Healthcare professionals: RR 0.88; 95% CI 0.79 to 0.97; six studies [39-42, 54, 56]; Tau² = 0.00; Chi² = 5.58, df = 5 (P = 0.35); I² = 10%			1. WHO/UNICEF materials: RR 0.66; 95% CI 0.43 to 1.01; four studies [40, 42, 46, 55]; Tau² = 0.00; Chi² = 1.44, df = 2 (P = 0.49); I² = 0%<br>2. Other materials: RR 0.89; 95% CI 0.79 to 1.00; three studies [41, 54, 56]; Tau² = 0.00; Chi² = 1.44, df = 2 (P = 0.49); I² = 0%
Wasting	RR 0.89; 95% CI 0.80 to 0.99; Tau² = 0.01; Chi² = 24.57, df = 10 (P = 0.006); I² = 59%<br>Seven studies [39, 40, 42, 52, 54, 56, 59]<br>11837 participants				
Weight gain (kg)	MD 0.04; 95% CI -0.02 to 0.10; Tau² = 0.00; Chi² = 9.43, df = 8 (P = 0.31); I² = 15%<br>Six studies [43, 48, 49, 54, 55, 60]<br>3576 participants				
Height gain (cm)	MD 0.07; 95% CI -0.11 to 0.24; Tau² = 0.02; Chi² = 11.03, df = 8 (P = 0.20); I² = 27%<br>Six studies [43, 48, 49, 54, 55, 60]<br>3574 participants				
Diarrheal disease	RR 1.24; 95% CI 0.58 to 2.63; Tau² = 0.38; Chi² = 11.31, df = 3 (P = 0.01); I² = 73%<br>Three studies [32, 41, 50]<br>929 participants				

Table S3: Supplementary feeding meta-analyses

Outcome	Composite analysis
Height-for-age Z-scores (HAZ)	MD 0.05; 95% CI -0.32 to 0.43; Tau² = 0.10; Chi² = 88.68, df = 2 (P < 0.00001); I² = 98%<br>Three studies [61-63]<br>3567 participants
Weight-for-age Z-scores (WAZ)	MD 0.44; 95% CI -0.03 to 0.92; Tau² = 0.16; Chi² = 172.96, df = 2 (P < 0.00001); I² = 99%<br>Three studies [61-63]<br>3567 participants
Weight-for-height Z-scores (WHZ)	MD 0.10; 95% CI -0.10 to 0.30; Tau² = 0.02; Chi² = 4.55, df = 2 (P = 0.10); I² = 56%<br>Three studies [61-63]<br>3567 participants
Stunting	RR 1.13; 95% CI 0.73 to 1.74; Tau² = 0.19; Chi² = 37.72, df = 5 (P < 0.00001); I² = 87%<br>Five studies [61-65]

	4732 participants
Wasting	RR 0.05; 95% CI 0.55 to 1.02; Tau² = 0.08; Chi² = 25.65, df = 4 (P < 0.0001); I² = 84%<br>Five studies [59, 61-63, 65]<br>7519 participants
Infant mortality	RR 0.61; 95% CI 0.38 to 0.97; Tau² = 0.00; Chi² = 0.35, df = 1 (P = 0.55); I² = 0%<br>Two studies [59, 65]<br>4757 participants

Table S4: Single study outcomes

Breastfeeding education	
Weight gain (kg)	MD 1.15 kg; 95% CI 0.95 to 1.35 [8]
Incidence of ARI	RR 0.55; 95% CI 0.42 to 0.72 [33]
Complementary feeding	
Infant mortality	RR 0.48; 95% CI 0.20 to 1.18 [59]
Underweight	RR 0.35; 95% CI 0.16 to 0.77 [42]
Change in weight-for-age (Z-scores)	MD 0.08; 95% CI -0.08 to 0.24 [53]
Change in weight-for-height (Z-scores)	MD 0.08; 95% CI -0.08 to 0.24 [53]
Supplementary feeding	
Diarrheal disease	RR 1.09; 95% CI 0.64 to 0.86 [63]
Weight gain (kg)	MD 0.15 kg; 95% CI -0.08 to 0.38 (50g soy-based); 36 participants<br>MD 0.29; 95% CI 0.06 to 0.52 (50g milk-based); 36 participants<br>MD 0.02 kg; 95% CI -0.17 to 0.21 (5g milk-based); 31 participants<br>MD 0.16 kg; 95% CI -0.17 to 0.21 (25g milk-based); 38 participants<br>MD 0.15 kg; 95% CI -0.09 to 0.39 (25g soy-based); 38 participants<br>MD 0.19 kg; 95% CI -0.12 to 0.50 (75g soy-based); 27 participants<br>MD 0.11lg; 95% CI -0.23 to 0.45 (75g milk-based); 27 participants[66]
Height gain (cm)	Not estimable (5g milk-based); 31 participants<br>MD 0.70 cm; 95% CI -0.10 to 1.50 (25g soy-based); 38 participants<br>MD 0.60 cm; 95% CI -0.29 to 1.49 (25g milk-based); 38 participants<br>MD 0.70 cm; 95% CI -0.19 to 1.59 (75g milk-based); 27 participants<br>MD 0.90 cm; 95% CI 0.06 to 1.74 (75g soy-based); 27 participants<br>MD 1.10 cm; 95% CI 0.28 to 1.92 (50g soy-based); 36 participants<br>MD 1.00 cm; 95% CI 0.27 to 1.73 (50g milk-based); 36 participants [66]
Change in weight-for-age	MD 0.20; 95% CI -0.10 to 0.50 (75g soy-based); 27 participants<br>MD 0.20; 95% CI -0.02 to 0.42 (25g milk-based); 38 participants<br>MD 0.00; 95% CI -0.21 to 0.21 (5g milk-based); 31 participants

	MD 0.10; 95% CI -0.13 to 0.33 (50g soy-based); 36 participants<br>MD 0.20; 95% CI -0.10 to 0.50 (75g milk-based); 27 participants<br>MD 0.20; 95% CI 0.00 to 0.40 (50g milk-based); 36 participants<br>MD 0.20; 95% CI -0.03 to 0.43 (25g soy-based) [66]
Change in height-for-age	MD 0.20; 95% CI -0.09 to 0.49 (25g milk-based); 38 participants<br>MD 0.20; 95% CI -0.01 to 0.41 (50g milk-based); 36 participants<br>MD 0.20; 95% CI -0.07 to 0.47 (75g milk-based); 27 participants<br>MD 0.20; 95% CI 0.20 to 0.45 (25g soy-based); 38 participants<br>MD 0.30; 95% CI -0.02 to 0.62 (75g soy-based); 27 participants<br>MD 0.10; 95% CI 95% CI -0.15 to 0.35 (5g milk-based); 32 participants<br>MD 0.30; 95% CI 0.04 to 0.56 (50g soy-based); 36 participants [66]
Change in weight-for-height	MD -0.10; 95% CI -0.43 to 0.23 (50g soy-based); 36 participants<br>MD 0.10; 95% CI -0.23 to 0.43 (50g milk-based); 36 participants<br>MD -0.10; 95% CI -0.38 to 0.18 (5g milk-based); 31 participants<br>MD 0.00; 95% CI -0.45 to 0.45 (75g milk-based); 27 participants<br>MD 0.00; 95% CI -0.40 to 0.40 (75g soy-based); 27 participants<br>MD 0.00; 95% CI -0.38 to 0.38 (25g soy-based); 38 participants<br>MD 0.00; 95% CI -0.27 to 0.27 (25g milk-based); 38 participants [66]

Table S5: Search strategy

EMBASE	1) "breast feeding"/de OR "infant feeding"/de OR "breast feed*":ti,ab OR breastfeed*:ti,ab OR breastfed:ti,ab OR "breast fed":ti,ab OR "complementary feed*":ti,ab OR "supplementary feed*":ti,ab OR "complementary (is this on its own - complementary feed* is therefore unnecessary?)<br><br>2) counselling:ti,ab OR intervention*:ti,ab OR "intervention strateg*":ti,ab OR "growth monitor*":ti,ab OR "mother* education":ti,ab OR education:ti,ab OR "prenatal care"/de OR "prenatal care":ti,ab OR "antenatal care":ti,ab OR (any more terms here?)<br><br>3) child*:ti,ab OR infan*:ti,ab OR newborn*:ti,ab OR neonate*:ti,ab OR baby:ti,ab OR babies:ti,ab OR kid:ti,ab OR kid*:ti,ab OR toddler*:ti,ab OR "preschool child"/de OR infant/exp OR toddler/de OR (more terms?)<br><br>4) 1 and 2 and 3<br><br>5) limit 4 to human
MEDLINE	1) "breast feeding"[mh:noexp] OR "breast feed*"[tiab] OR breastfeed*[tiab] OR "breast fed"[tiab] OR breastfed[tiab] OR "complementary feed*"[tiab] OR "supplementary feed*"[tiab] OR "complementary food*"[tiab] OR "supplementary food*"[tiab] OR weaning[mh] OR wean*[tiab] OR "infant feed*"[tiab] OR "young child feed*"[tiab] OR "infant food*"[tiab] OR "young child food*"[tiab](Probably a good idea to use proximity/adjacency operators here if you are using Medline - to retrieve infant feeding, feeding of infants etc)

	2) Counselling[mh:noexp](Counseling in MeSH - one L) OR counselling[tiab] OR intervention*[tiab] OR “intervention strateg*”[tiab] (not necessary as you have intervention on its own) OR “growth monitor*”[tiab] OR “mothers/education”[mh] OR “mother’s education”[tiab] OR education[tiab] OR “prenatal care”[mh] OR “prenatal care”[tiab] OR “antenatal care”[tiab] OR ante natal care”[tiab] OR “pre natal care”[tiab] OR counseling[tiab]<br><br>3) Infant[mh] (there are more specific MeSH terms for Infant such as Infant, Newborn, Infant, Low Birth Weight, Infant, Premature etc)OR “child, preschool”[mh] OR child*[tiab] OR infan*[tiab] OR newborn*[tiab] OR neonate*[tiab] OR baby[tiab] OR babies[tiab] OR kid[tiab] OR kid*[tiab] OR toddler*[tiab]<br><br>4) 1 and 2 and 3<br><br>5) limit 4 to humans (For both these searches there is no study designs filter which you state will be derived from the EPOC filters - this needs to be included here. Also - you state that the review will be concerning infants and under 2s in LMICs, but there is no LMICs filter included in these search strategies) These strategies are unfinished and more work needs to be done.

Table S6: Characteristics Table

Study name	Methods	Participants	Interventions	Outcomes	Notes
Agrasada 2011	**Exclusive Breastfeeding**<br><br>**Design:** Randomized controlled trial	**Location/Setting:** Philippine General Hospital in Manila, Phillippines<br><br>**Sample size:** 204 randomized<br><br>**Dropouts/withdrawals:** 25 lost to follow up noted<br><br>**Mean age:** infant age 3-5 days<br><br>**Inclusion criteria:** First-time mothers were eligible if they were 18 years or older, intended to breastfeed, and had vaginally delivered a low birth weight singleton with an Apgar score of 8 or higher at 5 min. The infant had to be born between 37 and 42 weeks of gestation, as computed from the mother’s last menstrual date and confirmed by Ballard scoring<br><br>**Exclusion criteria:** mothers taking medications that may compromise breastfeeding, and those who were not staying, together with their infants, in the study area until the infant was 6 months old.	Intervention (sample size)<br><br>Group 1 (n = 68)<br>Mothers received breastfeeding counselling<br><br>Group 2 (n = 67)<br>mothers received general child care counselling (without breastfeeding counselling)<br><br>**Dosage:** On eight occasions starting at infant age 3-5 days, then at 7-10 days, 21 days, 6 weeks and monthly thereafter till 22 weeks.<br><br>**Mode:** home visits<br>**Duration:** 22 weeks<br><br>**Given by:** breastfeeding counsellors<br><br>Control (n = 69)<br>Control mothers did not receive any counselling.	Weight (kg)<br><br>Height (cm)<br><br>Weight for age z-score<br><br>Infection (n)<br><br>Diarrhea (n)	**Study start date and end date:** January 2001 to August 2002<br><br>**Study duration:** 19 months<br><br>**Conflict of interest:** None to declare<br><br>**Source of funding:** This study was supported by research grants from the Swedish International Development Cooperation Agency (SIDA), In Develop and Uppsala University.
Ahmed 2008	**Exclusive Breastfeeding**<br><br>**Design:** Randomized controlled trial	**Location/Setting:** neonatal intensive care units (NICU) of three governmental and university hospitals in Cairo, Egypt<br><br>**Sample size:**60 infant mother pairs randomized<br><br>**Dropouts/withdrawals:** no lost to follow up was noted<br><br>**Mean age:** mean gestational age among infants of both groups was 32.2 ± 6.33 weeks<br><br>**Inclusion criteria:** The participants were a convenience sample of 60 mothers and their preterm infants who were born before 37 weeks of gestation and who were able and willing to breast feed their preterm infants<br><br>**Exclusion criteria:** not **specified**	Intervention (sample size)<br><br>Group 1 (n = 30 infant mother pairs) A five-session breastfeeding educational program<br><br>**Mode:** individual instruction sessions<br><br>**Given by:** researchers<br><br>**Duration:**1 month<br><br>Control (n = 30 mother infant pairs)<br><br>Routine care of the unit only	Exclusive breastfeeding (n)	**Study start date and end date:** not **specified**<br><br>**Study duration:** not **specified**<br><br>**Conflict of interest:** not **specified**<br><br>**Source of funding:** not **specified**
Aidam 2005	**Exclusive Breastfeeding**<br><br>**Design:** Randomized controlled trial	**Location/Setting:** in a Township in Tema, Ghana.<br><br>**Sample size:** 231 mothers randomized<br><br>**Dropouts/withdrawals:** 13 participants lost to follow up<br><br>**Mean age:** not **specified**<br><br>**Inclusion criteria:** Pregnant women in their last trimester of pregnancy, planning to deliver in the selected hospitals and to stay in Tema or Ashiaman for at least 6 months after delivery, were included. On delivery, term infants (36 – 44 week gestation) who were singletons, with normal birth weight (2500 g) and APGAR scores 6 at 1 and 5 minutes, were included.<br><br>**Exclusion criteria:** multiple birth, low Apgar score or planning to move out of areaParticipant characteristics:	Group 1 (n = 43)<br>EBF support given pre, peri, postnatally<br><br>Group 2 (n = 44)<br>EBF support given only peri and postnatally 2 visits prenatally and 9 visits until 6 months postpartum<br><br>**Mode:** education sessions and home visits<br><br>**Duration:** 6 months<br><br>**Given by:** Health personnel<br><br>Control (n = 49)<br>Nonbreastfeeding support health educational support	Exclusive breastfeeding up to 6 months (n)	**Study start date and end date:** 2002 to 2003<br><br>**Study duration:** 1 year<br><br>**Conflict of interest:** not **specified**<br><br>**Source of funding:** Funded by the University of Connecticut Research Foundation and LINKAGES USAID.
Aksu 2011	**Exclusive Breastfeeding**<br><br>**Design:** Randomized controlled trial	**Location/Setting:** in a Hanım Maternity Hospital located in Aydın, Turkey<br><br>**Sample size:** 66 infant-mother pairs randomized<br><br>**Dropouts/withdrawals:** 6/33 were lost to follow up in each intervention and the control group<br><br>**Mean age:** The gestational age at delivery was 39.2+1.3 weeks and 39.6 + 1.2 weeks in intervention and control groups respectively. The maternal age was 22.5+3.5 and 23.0 + 4.6 years in intervention and control groups respectively.	Intervention n = 33<br><br>Standard Breastfeeding education first few hours after delivery and at home on day 3 postpartum<br><br>**Mode:** home visits<br><br>**Given by:**breast feeding supporters<br><br>**Duration:** 5 months	Breastfeeding initiation in 1 hour and in 24 hour (n)<br><br>Exclusive breastfeeding (n)	**Study start date and end date:** between March and July 2008<br><br>**Study duration:** 5 months<br><br>**Conflict of interest:** not **specified**<br><br>**Source of funding:** not **specified**

		**Inclusion criteria:** Being primaparous (giving birth to a live infant for the first time), giving birth through the vaginal route, delivering a healthy newborn, birth occurring at the gestational age of 37 weeks or more, giving birth to a singleton baby, providing informed consent, living in the city of Aydın (to make home visits more convenient), being able to communicate/speak in Turkish, not using any drugs that would be likely to affect breast milk, having an intention to breastfeed, not having a history of chronic diseases, and not smoking were the factors that served as inclusion criteria.<br><br>**Exclusion criteria:** Infants lower than 2500 grams at birth, with an Apgar score of 7 or lower, with congenital anomalies or serious disease or those necessitating intensive care were excluded from the study	Control: Only standard breastfeeding education<br><br>n = 33		
Albernaz 2003	**Exclusive Breastfeeding**<br><br>**Design:** Randomized controlled trial	**Location/Setting:** in 3 hospitals in Pelotas, Brazil<br><br>**Sample size:** 188 mothers randomized<br><br>**Dropouts/withdrawals:** 16/94 in intervention group and 31/94 in control group were lost to follow up due to exclusion and withdrawal<br><br>**Mean age:** not **specified**<br><br>**Inclusion criteria:** The inclusion criteria was residence in the urban area of Pelotas, single birth, gestational age between 37 and 42 full weeks, lack of significant perinatal morbidity (postnatal stay at the intensive care unit should be 24 h), absence of maternal smoking, no economic constraints to growth (family income should be equal or superior to US$500/mo) and maternal intention to breast-feed.<br><br>**Exclusion criteria:** multiple birth, gestational age not 37-42 weeks, significant perinatal morbidity, maternal smoking and family income USD 500 per month	Intervention n = 94<br><br>Lactation counselling In addition to the hospital counselling visit, mothers were counselled at home when the infant was aged 5, 15, 30, 45, 60, 90 and 120 days.<br><br>**Mode:** counselling & home visit<br><br>**Duration:** 4 months<br><br>**Given by:** 2 registered nurses<br><br>Control n = 94<br><br>no lactation counselling	Exclusive breastfeeding (n)<br><br>Continued breastfeeding (n)	**Study start date and end date:** August 1999 to January 2000<br><br>**Study duration:** 6 months<br><br>**Conflict of interest:** not **specified**<br><br>**Source of funding:** International Atomic Energy Agency
Arifeen 2009	**Exclusive Breastfeeding**<br><br>**Design:** Randomized controlled trial	**Location/Setting:** in Outpatient facilities in Matlab subdistrict of Bangladesh<br><br>**Sample size:** 20 clusters of newborn infants under 5 in the study area randomized<br><br>**Dropouts/withdrawals:** 7% increase in the population was seen in the final analysis.<br><br>**Mean age:** Not **specified**<br><br>**Inclusion criteria:** not **specified**<br><br>**Exclusion criteria:** Catchment populations which received child and reproductive health services from the International Centre for Diarrhoeal Disease Research, Bangladesh	Intervention<br><br>n = 10 clusters (average cluster size 14529 people)<br><br>IMCI—health-worker training, health-systems improvements, and family and community activities<br><br>**Mode:** training and improvement activities<br><br>**Given by:** study teams<br><br>**Duration:** 7 years<br><br>Control:<br><br>N = 10 clusters (average cluster size 18285 people)	mortality (n)<br><br>infection (n)<br><br>diarrhea (n)	**Study start date and end date:** February 2002 to 2009<br><br>**Study duration:** 6 years<br><br>**Conflict of interest:** This paper is part of the Multi-Country Evaluation of integrated management of childhood illness Effectiveness, Cost and Impact (Multi country evaluation), which is coordinated by the Department of Child and Adolescent Health and Development of World health organization, the institution that promotes Integrated management of childhood illness implementation worldwide. All authors received support from World health organization/multi-country evaluation in terms of salaries, consultancies, and/or travel allowances.<br><br>**Source of funding:** Bill & Melinda Gates Foundation, World health organization's Department of Child and Adolescent Health and Development,<br><br>and United States Agency for International Development
Ayiasi 2016	**Exclusive Breastfeeding**<br><br>**Design:** Randomized controlled trial<br><br>Unit of randomization: At the level of health centres	**Location/Setting:** in 16 health centres in Masindi and Kiryandongo districts of Uganda<br><br>**Sample size:**1644 pregnant women<br><br>**Dropouts/withdrawals:** 122/879 in control group and 120/751 in intervention group were lost to follow up due to missing outcomes of interest or not found<br><br>**Mean age:** not **specified**<br><br>**Inclusion criteria:** not **specified**<br><br>**Exclusion criteria:** not **specified**	Intervention (n = 751)<br><br>Package of two closely linked components:<br><br>i) Village health teams making home visits to provide educational messages for maternal and newborn care<br><br>ii) each Village health teams was equipped with a mobile phone handset capable of making unlimited phone call consultation with professional health workers in case of further clarification or advice.<br><br>**Mode:** home visit & phone call<br><br>**Given by:** Village health team<br><br>**Duration:** 5 days<br><br>Control (n = 893)	Breastfeeding in first 1 hour and in 24 hours (n)<br><br>infection (n)	**Study start date and end date:** May/June 2013 to October/December 2014<br><br>**Study duration:** 17 months<br><br>**Conflict of interest:** No conflicts to declare<br><br>**Source of funding:** Directorate General for Development Cooperation (DGDC) of Belgium.
Baqui 2008	**Exclusive Breastfeeding**<br><br>**Design:** Randomized controlled trial	**Location/Setting:** in three rural sub-districts (upazilas; Beanibazar, Zakigan and Kanaighat of Sylhet district) of Bangladesh<br><br>**Sample size:** 24 clusters randomized (113,816 females)<br><br>**Dropouts/withdrawals:** 3787/36059 in home care arm, 4210/40159 in community care arm and 4147/37598 in comparison arm due to absent during survey, decline to participate, abortion or still births<br><br>**Mean age:** mothers aged 27.7, 27.9 and 27.8 years in home care, community care and control group respectively.	Intervention (n = 76218)<br><br>Group 1 (n = 36059)<br>In the home-care arm, female community health workers (one per 4000 population) identified pregnant women, made two antenatal home visits to promote birth and newborn-care preparedness, made postnatal home visits to assess newborns on the first, third, and seventh days of birth, and referred or treated sick neonates.<br><br>Group 2 (n = 40159)<br>Occured every 10 months<br><br>**Mode:** home visits	Neonatal mortality rate	**Study start date and end date:** 2003 to 2006<br><br>**Study duration:** 3 years<br><br>**Conflict of interest:** US was the programme manager for the saving newborn lives initiative in Bangladesh by Save the Children (US). The other authors declare that they have no confl ict of interest.<br><br>**Source of funding:** United States Agency for International Development and saving newborn lives programme by Save the Children (US) with a grant from Bill and Melinda Gates Foundation.

		**Inclusion criteria:** All married women of reproductive age (15–49 years) were eligible to participate<br><br>**Exclusion criteria:** not **specified**	**Duration:** 30 months<br><br>**Given by:** Community mobilizers<br><br>Control (n = 37598)		
Bauserman 2015	**Complementary Feeding**<br><br>**Design:** Randomized controlled trial	**Location/Setting:** in rural communities of Equateur Province in the Democratic Republic of Congo<br><br>**Sample size:** 222 infants randomized<br><br>**Dropouts/withdrawals:** 20/111 in intervention group and 27/111 control group were lost to fup due to death, moved or withdrawal. Reasons similar across groups<br><br>**Mean age:** 6 months<br><br>**Inclusion criteria:** We recruited 5-month-old infants who were exclusively breast-fed and whose mothers intended to continue breast-feeding for the first year of life<br><br>**Exclusion criteria:** We excluded infants who were likely to receive free or subsidized complementary foods formula, families likely to relocate during the study period, infants of multiple birth and infants with known congenital anomalies or neurological deficits	Intervention (n = 111 infants)<br>Given caterpillar cereal daily or the usual diet<br><br>**Mode:** Provided food<br><br>**Given by:** researchers and community health workers<br><br>**Duration:** 12 months<br><br>Control (n = 111 infants)	Stunting (n)<br><br>Height-for-age Z-scores<br><br>Weight-for- height Z-scoresWeight-for- age Z-scores	**Study start date and end date:** between 2010 and 2012<br><br>**Study duration:** 2 years<br><br>**Conflict of interest:** none to declare<br><br>**Source of funding:** supported by the Bill & Melinda Gates Foundation to FHI 360, through the Alive & Thrive Small Grants Program managed by the University of California at Davis and the Thrasher Research Fund.
Bhandari 2001	**Complementary Feeding**<br><br>**Design:** Randomized controlled trial	**Location/Setting:** in the urban slum of Nehru Place in Delhi, India<br><br>**Sample size:** 418 infants<br><br>**Dropouts/withdrawals:** 18/104, 8/104, 13/106 and 13/104 were lost to follow up in the food supplementation, nutrition counselling, no intervention and the vistation groups respectively.<br><br>**Mean age:** infants enrolled at 4 months of age and followed up until 12 months of age<br><br>**Inclusion criteria:** Four hundred and eighteen subjects were enrolled as they reached the age of 4 months if written informed consent was available<br><br>**Exclusion criteria:** Infants of families likely to emigrate during the study or with major congenital malformations were excluded.	Intervention<br>n = 312 children randomized<br>Group 1 (n = 104): a milk-based cereal and nutritional counseling<br><br>Group 2 (n = 104): monthly nutritional counseling alone.<br><br>Twice weekly. Then 2 at 4–5 months, 3 at 6–7 months, 4 at 8–9 months and 5 at 10–11 months of age.<br><br>**Mode:** packets<br><br>**Given by:** Researchers & nutritional counselling group<br><br>**Duration:** 10 to 11 months<br><br>Group 3 (n = 104): Only home visits<br>Received twice weekly home visits<br><br>Control: no intervention<br><br>n = 106	Weight (kg)<br><br>Height (cm)	**Study start date and end date:** not **specified**<br><br>**Study duration:** not **specified**<br><br>**Conflict of interest:** not **specified**<br><br>**Source of funding:** United Nations Children's Fund,
Bhandari 2003	**Exclusive Breastfeeding**<br><br>**Design:** Randomized controlled trial	**Location/Setting:** in state of Haryana, India, in communities located 3–5 km from the main highway<br><br>**Sample size:**1115 infants<br><br>**Dropouts/withdrawals:** 120/588 in intervention group and 115/527 in control group were lost to follow up due to relocation, withdrawal and death<br><br>**Mean age:** not **specified**<br><br>**Inclusion criteria:** Infants were enrolled if they lived locally and parental consent was given with being born in a study village within 9 months of start of intervention<br><br>**Exclusion criteria:** not **specified**	Intervention n = 588 families<br><br>**Mode:** Breastfeeding education<br><br>**Given by:** Health and nutrition workers<br><br>**Duration:** 9 months<br><br>Control n = 527 families	Initiation of breastfeeding in 3 hours (n)<br><br>Exclusive breastfeeding for 3 months 6 months (n)<br><br>Height-for-age Z scores<br><br>Diarrhea incidence (n)	**Study start date and end date:** Between Jan 1, 1998, and March 31, 2002<br><br>**Study duration:** 4 years<br><br>**Conflict of interest:** None to declare<br><br>**Source of funding:** The work was funded by the Department of Child and Adolescent Health and Development of World Health Organization
Bhandari 2004	**Complementary Feeding** intervention<br><br>**Design:** Randomized controlled trial	**Location/Setting:** in state of Haryana in India. The communities selected were located 3–5 km from the main highway<br><br>**Sample size:** 8 communities randomized 1025 infant-mother pairs<br><br>**Dropouts/withdrawals:** 117/552 in intervention group and 79/473 control group lost to follow up due to not available, refusal or death from both groups . Reasons similar across groups<br><br>**Mean age:** maternal age 23.6 years<br><br>**Inclusion criteria:** newborns enrolled if they were local residents and informed written consent was obtained<br><br>**Exclusion criteria:** not **specified**	Intervention n = 552<br><br>Promoted recognition of malnutrition problem with complementary feeding initiation at 6 months of age and appropriate portion size of feeds, optimal meal frequency, food density, and encouraging the child to eat.<br><br>**Mode:** home visits<br><br>**Given by:** Anganwadi workers, immunization clinics run by the auxiliary nurse midwives, and health care providers<br><br>**Duration:** 12 months<br><br>Control n = 473	Weight (kg)<br><br>Height (cm)<br><br>Height-for-age Z score<br><br>Weight for age Z score	**Study start date and end date:** 1998 to 2000<br><br>**Study duration:** 2 years<br><br>**Conflict of interest:** not **specified**<br><br>**Source of funding:** Department of Child and Adolescent health and Development, World Health Organization, Geneva, Switzerland.
Bolam 1998	**Exclusive Breastfeeding**<br><br>**Design:** randomized controlled trial	**Location/Setting:** in main maternity hospital in Kathmandu, Nepal.<br><br>**Sample size:** 540 mothers randomized<br><br>**Dropouts/withdrawals:** 41/135, 30/135, 39/135 and 37/135 were lost to follow up in the 4 groups respectively due to withdrawal and deaths<br><br>**Mean age:** 23.43 years<br><br>**Inclusion criteria:** All pregnant women admitted to Prasuti Griha hospital for delivery residing in these two communities were eligible for entry to the trial	Intervention (n = 295)<br><br>Postnatal health education for mothers on infant care and postnatal family planning practices.<br><br>Group A: (n = 135) immediately after birth and three months later<br><br>Group B: (n = 135) at birth only,<br><br>Group c: (n = 135) at three months only	Exclusive breastfeeding greater than or equal to 5 months (n)<br><br>Exclusive breastfeeding less than 5 months (n)	**Study start date and end date:** November 1994 to May 1996<br><br>**Study duration:** 3 years<br><br>**Conflict of interest:** None to declare<br><br>**Source of funding:** Research grant from Britain's Department for International Development.

		**Exclusion criteria:** mothers with still births	**Mode:** One on on health education<br><br>**Duration:** 18 months<br><br>**Given by:** Three female health educators, two midwives, and one community health worker were trained to give the health education.<br><br>Control (n = 135)<br><br>Group D: No education		
Cangöl 2017	**Exclusive Breastfeeding**<br><br>**Design:** Randomized controlled trial	**Location/Setting:** during a pregnancy preparation course in a state hospital located in Usak, western Turkey<br><br>**Sample size:** 100 pregnant women randomized<br><br>**Dropouts/withdrawals:** 16/50 in intervention and 17/50 in control group discontinued follow up<br><br>**Mean age:** The average ages of the pregnant women in the BMP and control groups were 22.62 ± 4.48 and 22.57 ± 4.33 years, respectively<br><br>**Inclusion criteria:** The inclusion criteria were being a primigravida (in their first pregnancy), being in the 32nd gestational week, being married, not working, having no physical disabilities, having no diagnosis of a psychological disorder, not experiencing a risky pregnancy, and not undergoing a planned cesarean section.<br><br>**Exclusion criteria:** not **specified**	Intervention (n = 50)<br><br>Breastfeeding motivation program (BMP)<br><br>The First BMP: between 32nd and 36th weeks in the antenatal period.<br><br>The Second BMP: on the first postnatal day.<br><br>The Third BMP: between the fourth and sixth postnatal weeks.<br><br>The Fourth BMP: in the fourth postnatal month.<br><br>**Mode:** Sessions<br><br>**Duration:** 5 months<br><br>**Given by:** researchers<br><br>Control (n =50)	Initiation of breastfeeding in 1 hour (n)	**Study start date and end date:** February and November 2014<br><br>**Study duration:** 10 months<br><br>**Conflict of interest:** None to declare<br><br>**Source of funding:** The authors received no financial support for the research, authorship, and/or publication of this article
Christian 2015	**Complementary Feeding**<br><br>**Design:** Randomized controlled trial	**Location/Setting:** in rural north-western Gaibandha & Union of the Rangpur district, under the 'JiVitA Project' of the Johns Hopkins University in Bangladesh.<br><br>**Sample size:** 596 clusters (5449 infants enrolled)<br><br>**Dropouts/withdrawals:** 382/5536 participants in 5 groups lost to follow up due to death, refusal and permanenetly moved . Reasons are similar across groups<br><br>**Mean age:** not **specified**<br><br>**Inclusion criteria:** not **specified**<br><br>**Exclusion criteria:** To ensure full exposure to a year of supplementation, per protocol, consented children who were not met despite repeated visits were excluded from the study once they had reached their 7-month birthday.	**Location/setting:** in rural north-western Gaibandha & Union of the Rangpur district, under the 'JiVitA Project' of the Johns Hopkins University in Bangladesh.<br><br>**Sample size:** 596 clusters (5449 infants enrolled)<br><br>**Dropouts/withdrawals:** 382/5536 participants in 5 groups lost to follow up due to death, refusal and permanently moved . Reasons are similar across groups<br><br>**Mean age:** not **specified**<br><br>**Inclusion criteria:** not **specified**<br><br>**Exclusion criteria:** To ensure full exposure to a year of supplementation, per protocol, consented children who were not met despite repeated visits were excluded from the study once they had reached their 7-month birthday.	Height-for-age Z-scores<br><br>Weight-for- height Z-scores<br><br>Weight for age Z-scores	**Study start date and end date:** 2012 to 2014<br><br>**Study duration:** 2 years<br><br>**Conflict of interest:** none to declare<br><br>**Source of funding:** supported by the United States Department of Agriculture, National Institute of Food and Agriculture (NIFA), under the Food and Nutrition Enhancement Program
De Oliveira 2006 (a)	**Supplementary Feeding**<br><br>**Design:** Randomized controlled trial	**Location/Setting:** in four municipalities of João Pessoa, Brazil<br><br>**Sample size:** 135 children<br><br>**Dropouts/withdrawals:** 3 lost to follow up from intervention group and 1 lost to follow up from control group<br><br>**Mean age:** not **specified**<br><br>**Inclusion criteria:** children attending in all municipal day care centres<br><br>**Exclusion criteria:** being in use of medicines as sulfate, vitamin supplements or other medication (n = 22), to be absent from the during six days (10.0% of the total study, n = 90) in the period of supplementation, or weight adequacy for age <-3Z-scores (n = 3)	intervention 1 (GI1 n = 48), intervention 2 (GI2 n = 45)<br><br>receiving 5g and 10g of the multi-mixture and placebo,<br><br>**Mode:** multi mixture supplement<br><br>**Given by:** single trained personnel<br><br>**Duration:** 2 months<br><br>Control (n = 42)	Malnutrition (n)<br><br>Stunting (n)<br><br>Wasting (n)	**Study start date and end date:** not **specified**<br><br>**Study duration:** not **specified**<br><br>**Conflict of interest:** none to declare<br><br>**Source of funding:** not **specified**
Fadnes 2016	**Exclusive Breastfeeding**<br><br>**Design:** Randomized controlled trial	**Location/Setting:** 5 years follow-up of the PROMISE-EBF trial in Mbale District, Eastern Uganda<br><br>**Sample size:** 765 infant-mother pairs<br><br>**Dropouts/withdrawals:** 154/396 in intervention group and 145/369 in control group were lost to follow up due to death and discontinuation<br><br>**Mean age:** median age of the mothers at the time of recruitment was 25 years (IQR 20–30)<br><br>**Inclusion criteria:** not **specified**<br><br>**Exclusion criteria:** not **specified**	Intervention n = 396<br><br>breast feeding counselling during the first 6 months of infancy<br><br>**Mode:** sessions<br><br>**Duration:** 6 months<br><br>**Given by:** peer counsellors<br><br>Control n = 369	Height-for-age Z-scores<br><br>Weight for age Z-scores	**Study start date and end date:** conducted between 2006 and 2011<br><br>**Study duration:** 6 years<br><br>**Conflict of interest:** None to declare<br><br>**Source of funding:** The study was part of the European Union-funded project PROMISE-EBF. It was also financially supported through the project 'Essential nutrition and child health in Uganda' funded by NUFU (Norwegian Programme for Development, Research and Education). LTF, IMSE, HS, NB, JVdB and TT were employed and funded by the University of Bergen. VN and JKT were employed and funded by Makerere University. SS and CL were employed and funded by Medical Research Council, South Africa
Flax 2014	**Exclusive Breastfeeding**<br><br>**Design:** Randomized controlled trial	**Location/Setting:** in Bauchi,Dass, and Ganjuwa local government areas of Bauchi State, Nigeria	Intervention (n = 229)	Initiation of breastfeeding in one hour and in 24 hours (n)	**Study start date and end date:** The baseline survey was conducted from August 2011 to November 2011, the intervention was implemented

		**Sample size:** 461 females<br><br>**Dropouts/withdrawals:** 33/229 in intervention group and 38/232 in control group were lost to follow up due to miscarriage, stillbirths, infant deaths etc<br><br>**Mean age:** mother's age is 25.6 years and child's age is 8.45 months<br><br>**Inclusion criteria:** micro credit clients were eligible to participate in the baseline survey if they were pregnant and aged 15–45 years.<br><br>**Exclusion criteria:** not **specified**	1. Trained credit officers led monthly breastfeeding learning sessions during regularly scheduled microcredit meetings for 10 months.<br><br>2. Text and voice messages were sent out weekly to a cell phone provided to small groups of microcredit clients (5–7 women). 3. The small groups prepared songs or dramas about the messages and presented them at the monthly microcredit meetings<br><br>**Mode:** Meetings, mobile, presentations<br><br>**Duration:** 10 months<br><br>**Given by:** Trained credit officers conducted learning sessions and distributed Federal Ministry of Health breastfeeding posters and leaflets during monthly microcredit meetings for 7 months<br><br>Control (n = 232)	Exclusive breastfeeding in 1 month and 6 months (n)	from November 2011 to August 2012, and the final survey took place from September 2012 to December 2012<br><br>**Study duration:** 17 months<br><br>**Conflict of interest:** V. L. Flax was a consultant to Partners for Development but did not consult or receive any payments for this project. M. Negerie, A. U. Ibrahim, S. Leatherman, E. J. Daza, and M. E. Bentley, no conflicts of interest<br><br>**Source of funding:** Supported by the Bill & Melinda Gates Foundation to Family Health International 360, through the Alive and Thrive Small Grants Program managed by the University of California, Davis, and Carolina Population Center grant R24HD050924
Froozani 1999	**Exclusive Breastfeeding**<br><br>**Design:** Randomized controlled trial	**Location/Setting:** in Maternity hospital in Shiraz, capital of Fars Province, the Islamic Republic of Iran<br><br>**Sample size:** 134 mother-infant pairs randomized<br><br>**Dropouts/withdrawals:** no lost to follow up was noted<br><br>**Mean age:** 23.2 years old<br><br>**Inclusion criteria:** mothers were primiparae or had previously been unsuccessful with breastfeeding, the pregnancy was normal and followed by vaginal delivery at term (38 ± 42 weeks), and the mothers had no chronic disease and were not taking any medication.<br><br>**Exclusion criteria:** not **specified**	Intervention (n = 59) (67 randomized)<br>Breastfeeding education<br><br>**Mode:** face-to-face, after delivery and during follow-up for 4 months in the mother and child health (MCH) centre or in their home<br><br>**Duration:** 7 months<br><br>**Given by:** Trained nutritionist<br><br>Control (n = 61) (67 randomized)	Exclusive breastfeeding at 1 month, 4 months (n)<br><br>Infection (n)<br><br>Diarrhea incidence (n)	**Study start date and end date:** March to September 1994<br><br>**Study duration:** 7 months<br><br>**Conflict of interest:** not **specified**<br><br>**Source of funding:** not **specified**
Grellety 2012	**Supplementary Feeding**<br><br>**Design:** Randomized controlled trial	**Location/Setting:** in villages of two districts of Maradi region in Niger<br><br>**Sample size:**2238 children<br><br>**Dropouts/withdrawals:** increase in participants in the survey<br><br>**Mean age:** not **specified**<br><br>**Inclusion criteria:** inclusion criteria of the MSF/Forsani distribution program<br><br>**Exclusion criteria:** not **specified**	Intervention (n = 1400)<br><br>Mass supplementation with ready to use supp RUSF<br><br>**Mode:** nutrition supplements<br><br>**Duration:** 4 months<br><br>**Given by:** researchers<br><br>Control (n = 838)	Wasting (n)<br><br>height (cm)<br><br>Height for age Z scores<br><br>Weight for height Z scores<br><br>mortality rate	**Study start date and end date:** July to October 2010<br><br>**Study duration:** 4 months<br><br>**Conflict of interest:** none to declare<br><br>**Source of funding:** Medecins Sans Frontires (MSF) and the United Nations International Children Emergency Fund (UNICEF)
Gu 2016	**Exclusive Breastfeeding**<br><br>**Design:** Randomized controlled trial	**Location/Setting:** in a tertiary hospital in Shanghai, China<br><br>**Sample size:** 352 females<br><br>**Dropouts/withdrawals:** 23/180 from intervention group and 44/172 from control group were lost to follow up<br><br>**Mean age:** 29.6 and 29.02 year old females in intervention and control group respectively.<br><br>**Inclusion criteria:** Chinese primiparous women who met the following criteria were included: (1) physically and mentally capable of communicating, reading and writing in Mandarin; (2) not having illnesses or problems that prohibit breast feeding for both mother and infant; (3) having attended at least one antenatal education class; and (4) accompanied with either a husband or a grandmother who met the following criteria as a significant other: (1) able to communicate and read in Mandarin; (2) having regular contact with the participant; and (3) willing and able to attend the intervention activities.<br><br>**Exclusion criteria:** not **specified**	Intervention (n = 180)<br><br>Mothers in the intervention group received individual instruction, group education, telephone counselling and routine nursing care in the postpartum period<br><br>**Mode:** TPB-based intervention programme<br><br>**Given by:** nurses<br><br>**Duration:** 4 months<br><br>Control (n = 172)<br><br>Routine nursing care only	Exclusive breastfeeding at 3 days, 6 weeks, 4 months, 6 months (n)	**Study start date and end date:** October 2013 to June 2014<br><br>**Study duration:** 9 months<br><br>**Conflict of interest:** None to declare<br><br>**Source of funding:** This work was supported by the Funding of Shanghai Science and Technology Committee
Guldan 2000	**Complementary Feeding**<br><br>**Design:** Randomized controlled trial	**Location/Setting:** in rural Sichuan county, China<br><br>**Sample size:** 495 infants<br><br>**Dropouts/withdrawals:** no lost to follow up was noted<br><br>**Mean age:** not **specified**<br><br>**Inclusion criteria:** pregnant women and women with infants up to 1 year old living in the study villages<br><br>**Exclusion criteria:** not **specified**	Intervention (n = 250)<br><br>monthly growth monitoring and counseling<br><br>**Mode:** home visits<br><br>**Given by:** nutrition counsellors<br><br>**Duration:** 12 months<br><br>Control (n = 245)	Height-for-age Z scores<br><br>Weight-for- height Z scoresWeight for age Z scores<br><br>Exclusive breastfeeding (n)	**Study start date and end date:** 1994 to 1995<br><br>**Study duration:** 1 year<br><br>**Conflict of interest:** none to declare<br><br>**Source of funding:** MISEREOR
Hanson 2015	**Exclusive Breastfeeding**<br><br>**Design:** Randomized controlled trial	**Location/Setting:** in six districts in Mtwara and Lindi regions, constituting rural areas in Southern Tanzania.<br><br>**Sample size:** 193,867 women interviewed at baseline (132 wards)<br><br>**Dropouts/withdrawals:** no lost to follow up was noted.<br><br>**Mean age:** not **specified**	Intervention (n = 65 wards)<br><br>home based counselling 3 home visits to women and their families during pregnancy and 2 visits in the first few days of the infant's life in 65 wards,<br><br>**Mode:** counselling sessions<br><br>**Given by:** 824 female volunteers	Breastfeeding immediately after delivery and in 1 hour (n)<br><br>mortality rate (n)	**Study start date and end date:** 2007 to 2013<br><br>**Study duration:** 6 years<br><br>**Conflict of interest:** The authors have declared that no competing interests exist.<br><br>**Source of funding:** This study received funding from the Bill & Melinda Gates Foundation through Saving Newborn Lives. The study also received

		**Inclusion criteria:** households in intervention and comparison wards with live births. If the village had fewer than 130 households, all households in the village were included<br><br>**Exclusion criteria:** If the village had greater than 130 households, segmentation was used to limit the sample to a maximum of 131 households	**Duration:** 37 months<br><br>Control (n = 67 wards)		funding from UNICEF Tanzania, the Batchworth Trust, and the Laerdal Foundation.
Hess 2015	**Supplementary Feeding**<br><br>**Design:** Randomized controlled trial	**Location/Setting:** in rural communities of the Dandé Health District in south western Burkina Faso<br><br>**Sample size:** 3220 children<br><br>**Dropouts/withdrawals:** 113/602, 114/613, 112/603, 136/617 from intervention groups and 119/785 from control group<br><br>**Mean age:** 9 months<br><br>**Inclusion criteria:** Children were considered eligible if they were 8.8 to 9.9 months of age, resided permanently in the area, planned to be available during the study period and had written parental consent.<br><br>**Exclusion criteria:** hemoglobin (Hb)<50 g/L, weight-for-length< 70% of the median of the National Center for Health Statistics/World Health Organization (NCHS/WHO) growth reference presence of bipedal edema, other severe illness warranting hospital referral, congenital abnormalities potentially interfering with growth, chronic medical conditions requiring frequent medical attention, known HIV infection of infant or mother, history of allergy towards peanuts, history of anaphylaxis or serious allergic reaction to any substance requiring emergency medical care, and concurrent participation in any other clinical trial.	Intervention (n = 2435)<br><br>Groups:<br>1) SQ-LNS without zinc, placebo tablet<br>2) SQ-LNS containing 5mg zinc, placebo tablet<br>3) SQ-LNS containing 10mg zinc, placebo tablet<br>4) SQ-LNS without zinc and 5mg zinc tablet given daily<br><br>**Mode:** tablet<br><br>**Duration:** 9 months<br><br>**Given by:** researchers<br><br>Control (n = 785)	Stunting (n)<br><br>Wasting (n)<br><br>Height (cm)	**Study start date and end date:** April 2010 to July 2012<br><br>**Study duration:** 28 months<br><br>**Conflict of interest:** none to declare<br><br>**Source of funding:** not **specified**
Iannoti 2017	**Complementary Feeding**<br><br>**Design:** Randomized controlled trial	**Location/Setting:** in communities of Cotopaxi Province, Ecuador<br><br>**Sample size:** 175 mothers randomized<br><br>**Dropouts/withdrawals:** 5/80 in intervention group and 10/83 in control group participants were lost to follow up due to unknown reasons or relocation. Reasons similar across groups<br><br>Children ages 6 to 9 months<br><br>**Inclusion criteria:** infant aged 6-9 months, singleton birth, and infant in good health<br><br>**Exclusion criteria:** congenital heart condition, severe acute malnutrition or egg allergy	Intervention (sample size)<br><br>Intervention group (n = 83)<br>Recieved food supplements and social marketing messages<br><br>**Dosage:** 1 egg per day for 6 months<br><br>**Mode:** home visits<br><br>**Duration:** 6 months<br><br>**Given by:** researchers<br><br>Control (n = 80)<br><br>No intervention + received social marketing messages	Height-for-age Z scores<br><br>Weight-for- height Z scoresWeight for age Z scores<br><br>diarrhoea (n)	**Study start date and end date:** March to December 2015<br><br>**Study duration:** 10 months<br><br>**Conflict of interest:** none to declare<br><br>**Source of funding:** Mathile institute for the Advancement of Human Nutrition
Ijumba 2015	**Exclusive Breastfeeding**<br><br>**Design:** Randomized controlled trial	**Location/Setting:** in a township community in Durban, South Africa<br><br>**Sample size:** 3957(120 per cluster)<br><br>**Dropouts/withdrawals:** 192/1821 in intervention group and 271/2136 in control group were lost to follow up.<br><br>**Mean age:** 23 years<br><br>**Inclusion criteria:** women >17 years old, live in the cluster, pregnant and intellectually capable of giving consent and willing to be visited by research team.<br><br>**Exclusion criteria:** not **specified**	Intervention (n = 1821)<br><br>Mothers received breastfeeding education and counselling<br><br>**Dosage:** Home visits on the following days; 2 visits during pregnancy, one in the first 48 h after delivery, then at 3–4 d, 10–14 d, 3–4 weeks and a final visit at 8–9 weeks.<br><br>**Mode:** home visits<br><br>**Duration:** 2 to 3 months<br><br>**Given by:** community health workers<br><br>Control (n = 2136)<br><br>One home visit during antenatal period and 2 postnatal at 4-6 and 10-12 weeks.	Exclusive breastfeeding immediately after birth and at 1 hour (n)	**Study start date and end date:** June 2008 to July 2011<br><br>**Study duration:** 3 years 2 months<br><br>**Conflict of interest:** none to declare<br><br>**Source of funding:** funded through a grant from Save the Children Federation, Inc. (USA) and by the South African Medical Research Council (sub-grant # 354).
Isanaka 2009	**Supplementary Feeding**<br><br>**Design:** Randomized controlled trial .	**Location/Setting:** in 12 villages in Maradi, Niger<br><br>**Sample size:** Overall sample size was 3533 children.<br><br>**Dropouts/withdrawals:** 25/1477 and 115/1689 lost to follow up from intervention group and control group respectively.<br><br>**Mean age:** children were 6 to 60 months of age. Mean maternal age was 26.6 ± 6.7 years<br><br>**Inclusion criteria:** Children between 6 and 60 mo of age during the follow-up period were eligible for inclusion<br><br>**Exclusion criteria:** children reaching 60 mo of age were removed from follow-up when no longer eligible.	Intervention (n = 1477)<br><br>The number of children with height and weight measurements in August, October, December and February was 3,166, 3,110, 2,936 and 3,026<br><br>one packet per day of ready-to-use therapeutic food (500kcal / day) given daily<br><br>**Mode:** packets<br><br>**Given by:** field teams of trained nutritional assistants and research nurses<br><br>**Duration:**3 months<br><br>Control (n = 1689)<br><br>The number of children with height and weight measurements in August, October, December and February was 1689, 1635, 1545 and 1574	Wasting (n)<br><br>Weight for Height Z score<br><br>Mortality (child/year)	**Study start date and end date:** August 2006 to March 2007<br><br>**Study duration:** 8 months<br><br>**Conflict of interest:** none to declare<br><br>**Source of funding:** none to declare
Jahan 2013	**Exclusive Breastfeeding**	**Location/Setting:** in two antenatal clinics in urban Dhaka, Bangladesh	Intervention (n = 192)	Breastfeeding initiation immediately after birth (n)	**Study start date and end date:** November 2007 to August 2008.

	**Design:** Randomized controlled trial .	**Sample size:** 384 females randomized<br><br>**Dropouts/withdrawals:** 42/192 each from intervention and control group were lost to follow up.<br><br>**Mean age:** 23.5 years<br><br>**Inclusion criteria:** Women at a gestational age of 24 weeks attending the government Maternal and Child Health Training Institute, Azimpur, and the Marie Stopes Clinic, Bashbari, Dhaka, were invited to participate in the study.<br><br>**Exclusion criteria:** Women with complications and special requirements were excluded	short-term nutrition education for a 3-month intervention period.<br><br>**Dosage:** 1 hour sessions<br><br>**Mode:** education sessions<br><br>**Given by:** investigators<br><br>**Duration:** 10 months<br><br>Control (n = 192)	Exclusive breastfeeding at 1 month (n)<br><br>weight gain at 6-7mo (kg)	**Study duration:** 10 months<br><br>**Conflict of interest:** None to declare<br><br>**Source of funding:** The National College of Home Economics Dhaka, the Maternal and Child Health Training Institute, Azimpur, and the Marie Stopes Clinic, Dhaka, supported the study
Khan 2013	**Exclusive Breastfeeding**<br><br>**Design:** Randomized controlled trial	**Location/Setting:** in Matlab community , a rural sub district 57 km southeast Dhaka, Bangladesh<br><br>**Sample size:** 3214 women<br><br>**Dropouts/withdrawals:** 531/1607 in intervention group and 515/1607 in control group were lost to fup due to refusal to particpiate, absence or death<br><br>**Mean age:** 25.99 years<br><br>**Inclusion criteria:** A pregnancy test was provided to women reporting a missed menstrual period, and those with a viable foetus <14 weeks gestation by ultrasound examination were invited to participate in the trial<br><br>**Exclusion criteria:** not **specified**	Intervention (n = 1607)<br><br>Exclusive breastfeeding counselling (BFC) intervention group: women received counselling in 8 visits: two during the last trimester of pregnancy, one within 7 days of delivery and 5 at monthly intervals up to 6 months after delivery.<br><br>**Mode:** Exclusive breastfeeding counselling<br><br>**Given by:** Counselling was provided by nine female workers recruited from the local community.<br><br>**Duration:** Not **specified**<br><br>Control (n = 1607)	Height for age z-scores<br><br>Weight for height z-scores<br><br>Weight for age z-scores	**Study start date and end date:** November 2001 to march 2007<br><br>**Study duration:** 5 years 5 months<br><br>**Conflict of interest:** none to declare<br><br>**Source of funding:** The MINIMat research study was funded by the icddr,b the United Nations Children's Fund, the Swedish International Development Cooperation Agency (Sida), the UK Medical Research Council (MRC), the Swedish Research Council, the Department for International Development (DfID), the Japan Society for the Promotion of Science (JSPS), the Child Health and Nutrition Research Initiative (CHNRI), Uppsala University and the US Agency for International Development. The International Atomic Energy Agency (IAEA) also partly supported the study.
Khresheh 2011	**Exclusive Breastfeeding**<br><br>**Design:** Randomized controlled trial	**Location/Setting:** in Al-Karak government hospital and Prince Ali Military hospital, Mutah, Karak, Jordan<br><br>**Sample size:** 140 females randomized<br><br>**Dropouts/withdrawals:** 27/72 in intervention group and 23/68 In control group were lost to follow up<br><br>**Mean age:** not **specified**<br><br>**Inclusion criteria:** All primiparous women who had given birth vaginally at Al-Karak government hospital and Prince Ali Military hospital were invited to participate<br><br>**Exclusion criteria:** Women with gestational age of less than 36 weeks, who were multiparous, who lived outside southern Jordan or who were not contactable by telephone after discharge were not eligible for the study	Intervention (n = 72)<br><br>One-to-one postnatal educational session and follow-up phone calls at two months and four months postpartum,<br><br>**Mode:** Breastfeeding education program<br><br>**Given by:** researchers who are experienced maternity nurses and mothers. Intervention<br><br>**Duration:** 9 months<br><br>Control (n = 68)	Exclusive breastfeeding at 6 months (n)<br><br>Infection (n)	**Study start date and end date:** August 2008 to September 2009<br><br>**Study duration:** 13 months<br><br>**Conflict of interest:** None to declare<br><br>**Source of funding:** This research was supported by grant from Mutah University
Kimani-Murage 2017	**Exclusive Breastfeeding**<br><br>**Design:** Randomized controlled trial	**Location/Setting:** in two slums of Nairobi, Kenya (Korogocho and Viwandani) where the African Population and Health Research Center (APHRC) runs the Nairobi Urban Health and Demographic Surveillance System (NUHDSS), covering close to 70,000 residents<br><br>**Sample size:** 5824 mother-infant pairs in pre-intervention study; 1110 mother-infant pair in intervention study<br><br>**Dropouts/withdrawals:** 22% pairs lost from intervention group and 17% lost from control group as written in the manuscript<br><br>**Mean age:** not **specified**<br><br>**Inclusion criteria:** The inclusion criteria included all pregnant women aged between 12 and 49 years old, who were resident within the defined study area and their respective babies (when born).<br><br>**Exclusion criteria:** The exclusion criteria included (a) women of reproductive age who gave birth before receiving the intervention.	Intervention (n = 521)<br><br>Home based nutrition and BF counselling<br><br>Scheduled visits were:<br><br>pregnancy - monthly until week 34, then weekly until delivery; mother and baby pairs – weekly in the first month then monthly until12 months.Frequency during the fifth month was biweekly to prepare mothers for complementary feeding.<br><br>**Mode:** Home visit<br><br>**Given by:** community health workers<br><br>**Duration:** not **specified**<br><br>Control (n = 581)	Exclusive breastfeeding at 2 months and at 6 months (n)	**Study start date and end date:** between 2012 and 2015<br><br>**Study duration:** 3 years<br><br>**Conflict of interest:** none to declare<br><br>**Source of funding:** study was funded by the Wellcome Trust, Grant No. 078530/Z/05/Z (Pre-intervention study) and Grant No. 097146/Z/11/Z (Intervention Study) and DANIDA, Grant No. IND0912010 (Comparison study). This research was also made possible through the generous funding for the NUHDSS by the Bill and Melinda Gates Foundation (Grant No. OPP1021893) and core funding for APHRC by The William and Flora Hewlett Foundation (Grant No. 2009–40510), and the Swedish International Cooperation Agency (Grant No. 2011001578). P.L.G. was supported by a British Academy mid-career fellowships.
Kirkwood 2013	**Exclusive Breastfeeding**<br><br>**Design:** Randomized controlled trial	**Location/Setting:** in 98 zones in seven districts in the Brong Ahafo Region, Ghana<br><br>**Sample size:**19,981 pregnant women originally recruited, 18,609 eligible pregnancies but 16,329 deliveries<br><br>**Dropouts/withdrawals:** 1141/9435 from intervention group and 1139/9174 from control group were lost to follow up<br><br>**Mean age:** 15- to 45-year-old pregnant women<br><br>**Inclusion criteria:** trial included all pregnancies that ended in a livebirth or stillbirth between November (the month after which Newhints training was completed), 2008, and December, 2009<br><br>**Exclusion criteria:** not **specified**	Intervention (n = 9435)<br><br>2 counselling home visits during pregnancy and three in the first week of life to promote essential newborn-care practices,<br><br>**Mode:** home based counselling<br><br>**Given by:** resident research field workers.<br><br>**Duration:** 13 months<br><br>Control (n = 9174)	Breastfeeding within 1 hour (n)<br><br>Exclusive Breastfeeding for 26-32 days (n)<br><br>Mortality rate	**Study start date and end date:** November 2008 to December 2009<br><br>**Study duration:** 13 months<br><br>**Conflict of interest:** None to declare<br><br>**Source of funding:** WHO, Bill & Melinda Gates Foundation, and UK Department for International Development.

Kupratakul 2010	**Exclusive Breastfeeding**<br><br>**Design:** Randomized controlled trial	**Location/Setting:** in antenatal care clinics at the Department of Obstetrics and Gynecology, King Chulalongkorn Memorial Hospital, Faculty of Medicine, Chulalongkorn University and Theptarin Hospital, Bangkok, Thailand<br><br>**Sample size:** 80 pregnant females randomized<br><br>**Dropouts/withdrawals:** 3/40 lost to follow up in intervention group and 4/40 lost from control group<br><br>**Mean age:** 28.3 years<br><br>**Inclusion criteria:** The eligible pregnant were those with more than 32 weeks' gestation, healthy, delivery of full-term healthy infants, no disease or contraindications to breastfeeding, no nipple abnormalities, and infants<br><br>**Exclusion criteria:** The authors excluded pregnant women with high-risk and multifetal pregnancies	Intervention (n = 40)<br><br>KSPES on antenatal education and postnatal support strategies<br><br>**Mode:** educational sessions<br><br>**Given by:** The KSPES program on antenatal education took about three hours by only the principal investigator.<br><br>**Duration:** 3 months<br><br>Control (n = 40)	exclusive breastfeeding at 1 month, 2 month, at 4 months, at 5 months, at 6 months (n)	**Study start date and end date:** January 2009 to September 2009<br><br>**Study duration:** 9 months<br><br>**Conflict of interest:** None to declare<br><br>**Source of funding:** not **specified**
Kuusipalo 2006	**Supplementary Feeding**<br><br>**Design:** Randomized controlled trial	**Location/Setting:** in Lungwena community, in Mangochi district of Malawi, southeastern Africa.<br><br>**Sample size:** 128 infants randomized<br><br>**Dropouts/withdrawals:** only 1 child was lost to follow up<br><br>**Mean age:** 6 to 17 months of age<br><br>**Inclusion criteria:** Infants 6 to 17 months of age were eligible to participate in the study, if their guardian gave a written informed consent and if they were underweight, as defined by a weight that was below the green area when plotted on the Malawian road to health card (corresponding approximately to a weight-for-age z score less than -2 of the World Health Organisation (WHO)-adopted.<br><br>**Exclusion criteria:** Infants were not eligible if they had any of the following: weight below 5.5 kg, weight for height z score less than -3, severe medical condition requiring hospitalization, adverse reaction within 30 minutes after a test dose of 15 g of FS, or likelihood to move out of study area during follow-up.	Intervention (n = 110 infants)<br><br>received for 12 weeks at home 1 of 8 food supplementation schemes: nothing, 5, 25, 50, or 75 g/day milk-based FS or 25, 50, or 75 g/day soy-based FS given daily<br><br>**Mode:** food supplements<br><br>**Given by:** counselled guardians<br><br>**Duration:** 12 weeks<br><br>Control (n = 18 infants)	mean blood Hb (g/L)<br><br>Height for age Z score<br><br>Weight for height Z score<br><br>Weight for age Z score	**Study start date and end date:** between November 2002 and March 2003<br><br>**Study duration:** 5 months<br><br>**Conflict of interest:** none to declare<br><br>**Source of funding:** Academy of Finland, Foundation for Paediatric Research in Finland, and Medical Research Fund of Tampere University Hospital
Lutter 2008	**Supplementary Feeding**<br><br>**Design:** Quasi-experimental study	**Location/Setting:** in communities of Santo Domingo in province of Pinchincha, Ecuador<br><br>**Sample size:** 634 infants at baseline<br><br>**Dropouts/withdrawals:** 16 children dropped out of the study due to infection<br><br>**Mean age:** 6-25 months<br><br>**Inclusion criteria:** All infants living in poor communities and receiving government health service<br><br>**Exclusion criteria:** not specified	Intervention<br><br>Group 1 (n = 338)<br><br>PANN 2000 program: 1) Education, 2) training of health worker in IYCF, 3) community participation, 4) provision of FCF (food), 5) monitoring & evaluation.<br><br>**Dosage:** 65g of daily nutrition supplements provided with 275 kcal/d<br><br>**Mode:** surveys & home visits<br><br>**Duration:** 11 months<br><br>**Given by:** researchers<br><br>Control (n = 296)	Anemia (n)<br><br>Height-for-age Z scores<br><br>Weight-for- height Z scores<br><br>Weight for age Z scores	**Study start date and end date:** 2000 to 2001<br><br>**Study duration:** 1 year<br><br>**Conflict of interest:** none to declare<br><br>**Source of funding:** Micronutrient Initiative
Mangani 2015	**Complementary Feeding**<br><br>**Design:** Randomized controlled trial	**Location/Setting:** in Lungwena and Malindi, Malawi<br><br>**Sample size:** 840 children<br><br>**Dropouts/withdrawals:** 24/209 21/212, 22/210 and 23/209 were lost to follow up due to death and drop out in the 4 groups respectively.<br><br>**Mean age:** 6.0275 months<br><br>**Inclusion criteria:** The inclusion criteria included age 5.50–6.50 months, residence in the study area, and informed consent from at least 1 authorized guardian.<br><br>**Exclusion criteria:** The exclusion criteria were weight for length (WFL) <80% of the World Health Organization (WHO) reference median or presence of oedema, severe illness war-ranting hospitalisation on the enrolment day, history of peanut allergy, concurrent participation in another clinical trial, and any symptoms of food intolerance within 30 min after ingesting a 5-gtest dose of LNS(either milk- or soy-based) used in the trial.	Intervention (n = 631 infants)<br><br>Received either 71 g day-1 of micronutrient fortified CSB, 54 g day-1 of micronutrient- fortified LNS with milk protein base (milk–LNS) or 54 g day-1 of micronutrient- fortified LNS with soy protein base (soy–LNS) between 6 and 18 months of age.<br><br>**Mode:** Given 2- 4 daily servings as supplements<br><br>**Given by:** mothers and guardians<br><br>**Duration:** 12 months<br><br>Control (n = 209 infants)<br><br>No supplemental complementary food given during the primary follow-up, but received a delayed supplementation with 71 g per day-fortified corn–soy flour between 18 and 30 months of age.	Stunting (n)<br><br>Height for age Z score<br><br>Weight for length Z score<br><br>Weight for age Z score	**Study start date and end date:** not **specified**<br><br>**Study duration:** 1 year<br><br>**Conflict of interest:** none to declare<br><br>**Source of funding:** supported by Academy of Finland (grants 200720, 108873, 111685 and 109796), Foundation for Pediatric Research in Finland, Medical Research Fund of Tampere University Hospital, and the American people through the support of the Office of Health, Infectious Disease, and Nutrition, Bureau for Global Health, United States Agency for International Development
Martinez 2018	**Complementary Feeding**<br><br>**Design:** Randomized controlled trial	**Location/Setting:** in one municipality, Tecpán (population 95000), in a settlement cluster of rural agricultural Kaqchikel Maya families of Guatemala.<br><br>**Sample size:** 324 children randomized<br><br>**Dropouts/withdrawals:** 28/324 children were lost to follow up<br><br>**Mean age:** 15-45 months at enrollment<br><br>**Inclusion criteria:** Subjects were eligible if they were aged 6–24 months with a length/height-for-age Z score (LAZ/HAZ) of less than or equal to −2.5 SD on WHO growth standards	Intervention (n = 161) growth monitoring, provision of multiple micronutrient powder supplement, a biweekly food ration and complementary feeding messages based on WHO recommendations<br><br>**Dosage:** monthly<br><br>**Mode:** education sessions<br><br>**Duration:** 6 months<br><br>**Given by:** Community health workers	Change in Height-for-age Z score	**Study start date and end date:** not **specified**<br><br>**Study duration:** 6 months<br><br>**Conflict of interest:** work was financially supported by a grant from GrandChallenges Canada to PR and MPG; BM, MFW and PR are current staff membersand KD is a former staff member at Maya Health Alliance, the partnering healthcare organisation for this study in Guatemala.

		**Exclusion criteria:** acute malnutrition (weight-for-length/height Z score(WLZ/WHZ) of less than or equal to −2 SD) or severe medical illness.	Control (n = 163) received usual care		**Source of funding:** supported by Grand Challenges Canada, grant numberSB-1726251050.
More 2012	**Exclusive Breastfeeding**<br><br>**Design:** Randomized controlled trial	**Location/setting:** in Slums in Mumbai, India<br><br>**Sample size:** 48 clusters randomized (18,197 births of which 18,039 were live and included)<br><br>**Dropouts/withdrawals:** 1499/9155 in intervention group and 1506/9042 in control group were lost to follow up<br><br>**Mean age:** not **specified**<br><br>**Inclusion criteria:** Clusters included had at least 1,000 households, residents were aware of no plans for resettlement, and cluster separation was wide enough to minimize contamination.<br><br>**Exclusion criteria:** We excluded areas with transient communities—large construction gangs, pavement dwellings—and areas for which resettlement was being negotiated	Intervention (n = 9082 live births)<br><br>Perinatal care, conduct meetings with women, attend planning and supervision meetings, and support group action. Through 36-meeting cycle<br><br>**Mode:** meetings<br><br>**Given by:** Sakhi (friend) was a local woman with secondary education and leadership skills, preferably married with children.<br><br>**Duration:** 6 months<br><br>Control (n = 8957 live births)	breastfeeding within 24 hours of birth (n)<br><br>Exclusive breastfeeding at 1 month (n)<br><br>Mortality	**Study start date and end date:** from 1st October 2006 to 30th September 2009<br><br>**Study duration:** 2 years 11 months<br><br>**Conflict of interest:** DO is a member of the PLoS Medicine Editorial Board. All authors have no other competing interests to declare.<br><br>**Source of funding:** The interventions involved in the City Initiative for Newborn Health were funded by the ICICI Foundation for Inclusive Growth – Centre for Child Health and Nutrition. Evaluative aspects of the trial were funded from 2007 by The Wellcome Trust. DO was funded by a Wellcome Trust Fellowship
Morrow 1999	**Exclusive Breastfeeding**<br><br>**Design:** Randomized controlled trial	**Location/Setting:** in a periurban community on the southwestern outskirts of Mexico City, Mexico<br><br>**Sample size:** 130 mothers randomized<br><br>**Dropouts/withdrawals:** 5/130 were lost to follow up<br><br>**Mean age:** not **specified**<br><br>**Inclusion criteria:** All pregnant women residing in the study area were considered eligible, visited athome by a study physician to verify eligibility, and invited toparticipate in a study of breastfeeding practices<br><br>**Exclusion criteria:** Women were considered ineligible and excluded from the study if they refused participation or moved out of the area before the first postpartum home visit, or if the baby died	Intervention 6 visits group (n = 44) and 3 visits group (n = 52)<br><br>Home based peer counselling for breastfeeding<br><br>1- Six-visit group: mothers were visited in mid and late pregnancy, in the first week and weeks 2, 4, and 8 post partum.<br><br>2- Three-visit intervention group: mothers were visited in late pregnancy, in the first week, and week 2 post partum.<br><br>**Mode:** home visits<br><br>**Given by:** Peer counsellors recruited from the same community and trained by La Leche League.<br><br>**Duration:** 4 months<br><br>Control N= 34	Initiation of breastfeeding within 1 hour and within 24 hours (n)<br><br>Exclusive breastfeeding for 3 months (n)	**Study start date and end date:** march 1995 to December 1996<br><br>**Study duration:** 22 months<br><br>**Conflict of interest:** not **specified**<br><br>**Source of funding:** study was supported by research grants from Wellstart International's Expanded Promotion of Breastfeeding Program (USAID) cooperative agreement DPE-5966-A-00-1045-00) and the US National Institute of Child Health and Human Development(HD13021).
Nair 2017	**Exclusive Breastfeeding**<br><br>**Design:** Randomized controlled trial	**Location/Setting:** in two adjoining districts of Jharkhand and Odisha, India<br><br>**Sample size:** 5,781 pregnant females (120 clusters)<br><br>**Dropouts/withdrawals:** 92% of both arms were evaluated at the end<br><br>**Mean age:** not **specified**<br><br>**Inclusion criteria:** Individual participants were pregnant women identified and recruited in the study clusters and their children<br><br>**Exclusion criteria:** We excluded stillbirths and neonatal deaths, infants whose mothers died, those with congenital abnormalities, multiple births, and mother and infant pairs who migrated out of the study area permanently during the trial period	Intervention n = 2814 mothers<br><br>Worker carried out one home visit in the third trimester of pregnancy, monthly visits to children younger than 2 years to support feeding, hygiene, care, and stimulation, as well as monthly women's group meetings to promote individual and community action for nutrition.<br><br>**Mode:** home visits<br><br>**Given by:** intervention team<br><br>**Duration:** 2 years<br><br>Control n =2967 mothers	Initiation of breastfeeding in 1 hour and exclusive breastfeeding (n)	**Study start date and end date:** Oct 2013 to June 2017<br><br>**Study duration:** 3 years 8 months<br><br>**Conflict of interest:** declare no competing interests<br><br>**Source of funding:** UK Medical Research Council, Wellcome Trust, UK Department for International Development (DFID)
Navarro 2013	**Exclusive Breastfeeding**<br><br>**Design:** Quasi experimental study	**Location/Setting:** in eight geographical areas of intervention in Sao Paulo, Brazil<br><br>**Sample size:** 603 Dyads<br><br>**Dropouts/withdrawals:** 73/266 dyads were lost to follow up from intervention group and 78/337 lost to follow up from control group<br><br>**Mean age:** 20.03 months<br><br>**Inclusion criteria:** groups of pregnant women which met every fifteen days according to proto-cols defined in ten educational meetings on health and nutrition during pregnancy.<br><br>**Exclusion criteria:** not **specified**	Intervention n = 266 dyads<br><br>A program that promotes key practices of maternal and child care through meetings with pregnant women and home visits to promote child growth and development was designed and implemented. Home visits each month and participated in a group activity held biweekly during pregnancy and monthly after birth.<br><br>**Mode:** home visits<br><br>**Duration:** 5 months<br><br>**Given by:** lay counsellors<br><br>Control n = 337 dyads	Exclusive breastfeeding 1 month (n)<br><br>Height for age Z scores<br><br>Diarrhea in last 2 weeks (n)	**Study start date and end date:** April 2005 to September 2007<br><br>**Study duration:** 2 years 6 months<br><br>**Conflict of interest:** None to declare<br><br>**Source of funding:** The intervention and the impact assessment were carried out with financial support from the United Nations Children's Fund (UNICEF), the Ministry of Public Health of the Dominican Republic, private donors from the Netherlands, and the partnership Action for Family Health (Catholic Medical Mission Board, Pan-American Health Organization (PAHO/WHO) , Brystol-Myers Squibb Foundation)
Ochola 2013	**Exclusive Breastfeeding**<br><br>**Design:** Randomized controlled trial	**Location/Setting:** in Kibera slum, Nairobi, Kenya<br><br>**Sample size:**360 females<br><br>**Dropouts/withdrawals:** 33/120 , 31/120 and 31/120 were lost to follow up from intervention arms and control group respectively<br><br>**Mean age:** 24.45 years<br><br>**Inclusion criteria:** The inclusion criteria were: (i) in the third trimester of pregnancy, 34–36 weeks' gestation; (ii) HIV negative; (iii) intention to stay in Kibera for at least 6 months after delivery; (iv) willing to be visited at home; (v) absence of documented chronic diseases such as diabetes mellitus, renal disease, heart dis-ease or any other chronic disease, and no eclampsia in a previous pregnancy; and (vi) willing to be included in the study.	Intervention Group 1 (n = 54)<br><br>The home-based intensive counselling group (HBICG): 7 counselling sessions at home by trained peers, one prenatally and 6 postnatally.<br><br>Intervention Group 2 (n = 62)<br><br>The facility based semi-intensive counselling group (FBSICG): one counselling session prenatally.<br><br>**Mode:** counselling	Exclusive breastfeeding for 6 months (n)	**Study start date and end date:** April 2006 to April 2008<br><br>**Study duration:** 2 years<br><br>**Conflict of interest:** None to declare<br><br>**Source of funding:** study was supported by the Nestle Foundation in its entirety

		**Exclusion criteria:** documented chronic diseases such as diabetes mellitus, renal disease, heart disease or any other chronic disease, and eclampsia in a previous pregnancy	**Given by:** Three females with a minimum of secondary level of education and residing in the study area were recruited as breast-feeding counsellors.<br><br>**Duration:** 5 months post-partum<br><br>Control n = 42		
Oelefse 2003	**Complementary Feeding**<br><br>**Design:** Randomized controlled trial	**Location/Setting:** in black urban disadvantaged community in the Western Cape Town, South Africa<br><br>**Sample size:** 46 children<br><br>**Dropouts/withdrawals:** 16/46 enrolled participants were lost to follow up due to leaving study area. Reasons similar across groups<br><br>**Mean age:** 6-15 months at baseline<br><br>**Inclusion criteria:** From the community, 60 children aged approximately 6 months were randomly se-lected from all mothers visiting the localclinic with their infants<br><br>**Exclusion criteria:** not **specified**	Intervention (n = 25)<br><br>multi-micronutrient fortified complementary food<br><br>**Mode:** complementary food<br><br>**Given by:** research assistants<br><br>**Duration:** 6 months<br><br>Control (n = 21)	Height-for-age Z-scores<br><br>Weight-for- height Z-scores<br><br>Weight for age Z-scores	**Study start date and end date:** not **specified**<br><br>**Study duration:** 1 year<br><br>**Conflict of interest:** not **specified**<br><br>**Source of funding:** not **specified**
Olaya 2013	**Complementary Feeding**<br><br>**Design:** Randomized controlled trial	**Location/Setting:** in 2 hospitals in Bogota, Colombia (Fontibon and Suba), that serve populations with low socio-economic status<br><br>**Sample size:** 85 infants at baseline<br><br>**Dropouts/withdrawals:** 85 randomly assigned with 76 completing the analysis.<br><br>**Mean age:** 6 months and followed up to 12 months of age<br><br>**Inclusion criteria:** Mothers of healthy term infants with birth weight >2500g who were participating in the growth-monitoring program at 2 hospitals in Bogota, Colombia (Fontibon and Suba), and who were exclusively breastfeeding when their infants were 4 months of age were approached and given information about the study<br><br>**Exclusion criteria:** not meeting above criteria or infants with a haemoglobin concentration of 11 g/dL (the cutoff used to define anaemia in Colombia)	Intervention (n = 38 infants)<br><br>intervention group (new guidelines group) NGG + counselling to:<br><br>1) continue breastfeeding 2) offer red meat 3 d/wk 3) offer fruit and vegetables daily<br><br>**Mode:** Food provision with education<br><br>**Given by:** mothers and researchers<br><br>**Duration:** 6 months<br><br>Control (n = 38 infants)	Linear growth (cm)<br><br>Hemoglobin level (g/dl)	**Study start date and end date:** not **specified**<br><br>**Study duration:** 1 year<br><br>**Conflict of interest:** none to declare<br><br>**Source of funding:** Childhood Nutrition Research Centre, University College London Institute of Child Health, and Pontificia Universidad Javeriana
Penfold 2014	**Exclusive Breastfeeding**<br><br>**Design:** Randomized controlled trial	**Location/Setting:** in six districts of Southern Tanzania<br><br>**Sample size:** 4986 women<br><br>**Dropouts/withdrawals:** only one ward lost to follow up from control group<br><br>**Mean age:** not **specified**<br><br>**Inclusion criteria:** not **specified**<br><br>**Exclusion criteria:** not **specified**	Intervention (n = 2486)<br><br>Promote recommended newborn care practices including hygiene, breastfeeding and identification and extra care for low birthweight babies.<br><br>3 home visits during pregnancy and 2 in the early neonatal period, with additional visits for small babies<br><br>**Mode:** home visits<br><br>**Given by:** 800 women volunteered<br><br>**Duration:** 12 months<br><br>Control (n = 2490)	Breastfeeding initiation in 1 hour (n)	**Study start date and end date:** not **specified**<br><br>**Study duration:** not **specified**<br><br>**Conflict of interest:** none to declare<br><br>**Source of funding:** The study was funded by the Bill & Melinda Gates Foundation through the Saving Newborn Lives program of Save the Children
Penny 2005	**Complementary Feeding**<br><br>**Design:** Randomized controlled trial	**Location/Setting:** in a community in Trujillo, Peru with a population of 600 000.<br><br>**Sample size:** 377 infants<br>**Dropouts/withdrawals:** no lost to follow up<br><br>**Mean age:** 0.15 days at birth<br><br>**Inclusion criteria:** newborns who were found at home, who were aged ≤ 10 days, who had no known congenital malformation or chronic condition that could affect growth, and whose parents gave written informed consent<br><br>**Exclusion criteria:** the main reasons for infants not being enrolled were that the needed sample size had been achieved or that the baby had been born before predicted and was outside the age criterion. Also excluded congenital malformation or chronic conditions that could affect growth of the baby. Health facilities excluded if the randomisation resulted in a control site being directly adjacent to an intervention site.	Intervention (n = 187)<br><br>educational sessions given<br><br>**Mode:** education sessions<br><br>**Given by:** researchers<br><br>**Duration:** 18 months<br><br>Control (n = 190)	Height-for-age Z-scores<br><br>Weight for age Z-scores<br><br>Weight for height Z-scores	**Study start date and end date:** not **specified**<br><br>**Study duration:** 18 months<br><br>**Conflict of interest:** none to declare<br><br>**Source of funding:** supported by the Family Health and Child Survival Cooperative Agreement between the United States Agency for International Development and Department of International Health, Johns Hopkins Bloomberg School of Public Health, MD, USA
Raeisi 2014	**Exclusive Breastfeeding**<br><br>**Design:** Randomized controlled trial	**Location/Setting:** in family health research centre in Vali-E-AsrHospital, Tehran, Iran<br><br>**Sample size:** 100 fathers<br><br>**Dropouts/withdrawals:** no lost to follow up<br><br>**Mean age:** not **specified**<br><br>**Inclusion criteria:** the inclusion criteria was as follows:<br><br>1)Healthy mother with no underlying disease.<br>2)Healthy mother with no pregnancycomplication.<br>3)Being in the second trimester of pregnancy	Intervention (n = 50)<br><br>The case group consisted of fathers attending training courses of breastfeeding during pregnancy<br><br>The courses were held three times from the 30th week of gestation to the end of pregnancy in a family health research center.<br><br>**Mode:** educational courses<br><br>**Given by:** The case group was provided with an educational package on promoting fathers' participation. They attended three training sessions where they were trained by brochures.	Exclusive breastfeeding at 6 months (n)<br><br>Infection (n)	**Study start date and end date:** not **specified**<br><br>**Study duration:** not **specified**<br><br>**Conflict of interest:** not **specified**<br><br>**Source of funding:** Tehran University of Medical Sciences- maternal, fetal and neonatal research center.

		**Exclusion criteria:** not **specified**	**Duration:** 7 to 8 weeks<br><br>Control (n = 50)		
Roy 2007	**Complementary Feeding**<br><br>**Design:** Randomized controlled trial	**Location/setting:** in 121 Community Nutrition Centers (CNCs) of the Bangladesh Integrated Nutrition Project (BINP) in four regions of Bangladesh<br><br>**Sample size:** 611 children<br><br>**Dropouts/withdrawals:** 35/611 were lost to follow up due to withdrawal, migration and death. Reasons similar across groups<br><br>**Mean age:** not **specified**<br><br>**Inclusion criteria:** Gomez Classification was used<br><br>**Exclusion criteria:** not **specified**	Intervention (n = 306)<br><br>weekly nutrition education<br><br>**Mode:** education sessions<br><br>**Given by:** Community health workers/counsellors<br><br>**Duration:** 6 months<br><br>Control (n = 305)	Height-for-age Z-scores<br><br>Weight for age Z-scores<br><br>Weight for height Z-scores	**Study start date and end date:** 2000 to 2002<br><br>**Study duration:** 2 years<br><br>**Conflict of interest:** none to declare<br><br>**Source of funding:** Bangladesh Integrated Nutrition Project (BINP), the Ministry of Health and Family Welfare, and the World Bank.
Saleem 2014	**Complementary Feeding**<br><br>**Design:** Randomized controlled trial	**Location/Setting:** in a community of a peri-urban setting of Karachi, Pakistan<br><br>**Sample size:** 212 infants<br><br>**Dropouts/withdrawals:** 23/118 from intervention and 19/94 from control group<br><br>**Mean age:** 10-20 weeks infants<br><br>**Inclusion criteria:** The study population comprises mothers of infants aged 10-20 weeks, who were either exclusively or partially breast fed but had not started CF or had recently started (less than one week prior to enrolment),and lived in the study area<br><br>**Exclusion criteria:** Infants were excluded if they were already below the 5th percentile in WHO growth charts on weight-for-age at baseline, had a history of two or more hospital admissions at the time of enrolment (each hospital stay >7 days),had serious congenital anomalies (cleft palate, congenital heart disease, neural tube defect), other chronic conditions impairing feeding (e.g. cerebral palsy) or the presence of acute illness, and/or severe anaemia, which required urgent hospitalization at the time of enrolment.	Intervention (n = 118 infants)<br><br>There were a total of four visits(baseline and three subsequent visits at 10 weeks interval<br><br>**Mode:** educational sessions<br><br>**Given by:** research trainees<br><br>**Duration:** 7 to 8 months<br><br>Control (n = 94 infants)	Stunting (n)<br><br>Wasting (n)<br><br>Malnutrition (n)	**Study start date and end date:** not **specified**<br><br>**Study duration:** 30 weeks<br><br>**Conflict of interest:** none to declare<br><br>**Source of funding:** funded by Aga Khan University Research Council and NIH-Fogarty research training fund
Santos 2001	**Complementary Feeding**<br><br>**Design:** Randomized controlled trial	**Location/Setting:** in 28 municipal health centres in the city of Pelotas, Brazil<br><br>**Sample size:** 424 children<br><br>**Dropouts/withdrawals:** 20/424 were lost to follow up<br><br>**Mean age:** not **specified**<br><br>**Inclusion criteria:** children attending the health centres were selected<br><br>**Exclusion criteria:** not **specified**	Intervention (n = 218)<br><br>nutrition-counselling component of the Integrated Management of Childhood Illnesses (IMCI) strategy,<br><br>**Mode:** doctors<br><br>**Given by:** researchers<br><br>Intervention **Duration:** not **specified**<br><br>Control (n = 206)	weight gain (kg)<br><br>height gain (cm)<br><br>Weight for age Z-scores	**Study start date and end date:** not **specified**<br><br>**Study duration:** 6 months<br><br>**Conflict of interest:** not **specified**<br><br>**Source of funding:** World Health Organization Department of Child and Adolescent Health
Santos 2005	**Complementary Feeding**<br><br>**Design:** Non-randomized study	**Location/Setting:** in a community of Alagoas in Northeast Brazil.<br><br>**Sample size:** 191 children<br><br>**Dropouts/withdrawals:** 19/191 were lost to follow up due to relocation or death. Reasons similar across groups<br><br>**Mean age:** 11.64 months<br><br>**Inclusion criteria:** In the 20 selected municipalities, mother s and their 6-18-month-old children attending primary health care facilities and followed-up by community health agents were identified. Ineach high-coverage municipality, the first 10children entering the Milk Program or already enrolled in it for as long as one month were selected for the "intervention" (supplementation) group.<br><br>**Exclusion criteria:** exclusion due to insufficient amount of supplement available at the municipal level to include all local children who needed it.	Intervention (n = 99)<br><br>Milk supplement feeding<br><br>**Mode:** supplements<br><br>**Given by:** researchers<br><br>**Duration:** 6 months<br><br>Control (n = 92)	Weight (g)<br><br>Height (cm)<br><br>height-for-age Z-scores<br><br>weight-for- height Z-scores<br><br>weight for age Z-scores	**Study start date and end date:** not **specified**<br><br>**Study duration:** 6 months<br><br>**Conflict of interest:** not **specified**<br><br>**Source of funding:** Brazilian ministry of health and the International atomic energy agency
Schroeder 2002	**Supplementary Feeding**<br><br>**Design:** Randomized controlled trial	**Location/Setting:** in a community of Phu Tho Province, west of Hanoi, Vietnam<br><br>**Sample size:** 238 children<br><br>**Dropouts/withdrawals:** 5/119 lost in intervention group and 1/119 lost in control group<br><br>**Mean age:** 15.5 months old<br><br>**Inclusion criteria:** used the following selection criteria: not currently enrolled in the longitudinal study; resident of one of the 34 hamlets in the six intervention communes; attending NERPs according to the latest list available; and age 6.0 to 23.9 months at baseline.<br><br>**Exclusion criteria:** Excluded multiple births or children with severe medical problems, such as handicap or measles	Intervention (n = 119) preparatory activities, training, situation analysis, and implementation<br><br>**Dosage:** not **specified**<br><br>**Mode:** sessions<br><br>**Duration:** not **specified**<br><br>**Given by:** Save the children staff members<br><br>Control (n =119)	Height-for-age Z-scores<br><br>Weight-for- height Z-scores<br><br>Weight for age Z-scores	**Study start date and end date:** December 1999 to December 2000<br><br>**Study duration:** 1 year<br><br>**Conflict of interest:** not specified<br><br>**Source of funding:** LINKAGES: Breastfeeding, LAM, Complementary Feeding, and Maternal Nutrition Program. LINKAGES is supported by G/PHN/HN, Global, the United States Agency for International Development (USAID
Schwartz 2015	**Exclusive Breastfeeding**<br><br>**Design:** Randomized controlled trial	**Location/Setting:** in a rooming-in facility of Hospital de Clínicas de PortoAlegre (HCPA). HCPA is a public general hospital in Porto Alegre, Brazil, and a Baby-friendlyHospital accredited facility<br><br>**Sample size:** 323 mothers	Intervention (n = 163) Mothers and grandmothers in the intervention group received counselling sessions on BF and healthy complementary feeding at the maternity ward and at home (7, 15, 30, 60, and 120 days after delivery). In the no-cohabitation group, adolescent mothers alone received the intervention. In the	BMI-for-age Z-scores<br><br>Height-for-age Z-scores	**Study start date and end date:** May 2006 to July 2013<br><br>**Study duration:** 7 years

		**Dropouts/withdrawals:** Of the 323 mothers/children who started the trial, 207(64.1%) took part in final assessment.<br><br>**Mean age:** 17.45 years mothers at baseline<br><br>**Inclusion criteria:** age younger than 20 years, lived within Porto Alegre municipal limits, had given birth to a healthy singleton infant with a birth weight of 2,500 g or greater, and had begun BF.<br><br>**Exclusion criteria:** Mothers of multiple infants, those who could not room in with their infants due to maternal or neonatal complications, and those who lived with their mothers-in-law (i.e., the child's paternal grandmother) were excluded from the study	cohabitation group, both mother and grand-mother received initial counselling; the initial session was held separately for mothers and grandmothers, on a one-on-one basis.<br><br>**Dosage:** maternity ward and at home (7, 15, 30, 60, and 120 days after delivery)<br><br>**Mode:** sessions<br><br>**Duration:** 4 months<br><br>**Given by:** Sessions were led by members of a team composed of two nurses, a dietitian, and a paediatrician, three of whom were International Board Certified Lactation Consultants (IBCLCs)<br><br>Control (n = 160)		**Conflict of interest:** not **specified**<br><br>**Source of funding:** Financial support was provided by FIPE-<br><br>HCPA (Research and Events Support Fund at Hospital de Clínicas de Porto Alegre) and CNPq (National Council for Scientific and Technological Development)
Shi 2009	**Complementary Feeding**<br><br>**Design:** Randomized controlled trial	**Location/Setting:** in Eight townships in Laishui, a rural area in China,<br><br>**Sample size:** 599 infants<br><br>**Dropouts/withdrawals:** 38/294 from intervention group and 71/305 from the control group<br><br>**Mean age:** 2-4 months old<br><br>**Inclusion criteria:** All infants in the selected townships who were full-term(gestational age >37 weeks), singletons, without major birth defects, and aged 2–4 months at the time of the baseline survey were eligible for the study<br><br>**Exclusion criteria:** not **specified**	Intervention (n = 294)<br><br>four major components: (i) group training sessions on food selec-tion, preparation and hygiene, childhood nutrition and growth, and responsive feeding style; (ii) demonstration of preparing enhanced weaning food recipes which we reformulated using locally available, affordable, acceptable and nutrient-dense foods such as egg, tomato, beans,meat, chicken and liver; (iii) booklets which contained infant feeding guidance and methods of preparing the recommended recipes; and (iv) home visits every three months to identify possible feeding problems and provide individual counselling<br><br>**Dosage:** every 3 months<br><br>**Mode:** educational sessions<br><br>**Given by:** local health-care providers,<br><br>**Duration:** 6 months<br><br>Control (n = 305)	Weight (kg)<br><br>Height (cm)	**Study start date and end date:** April 2006 to September 2007<br><br>**Study duration:** 18 months<br><br>**Conflict of interest:** none to declare<br><br>**Source of funding:** Proctor & Gamble Fellowship provided through the Johns Hopkins Bloomberg School of Public Health
Sikander 2015	**Exclusive Breastfeeding**<br><br>**Design:** Randomized controlled trial	**Location/Setting:** in 40 Union Councils of a rural district in the Mansehra district, located in the Khyber Pakhtunkhwa Province of Pakistan<br><br>**Sample size:** 454 pregnant females<br><br>**Dropouts/withdrawals:** 14/224 from intervention group and 17/228 from control group were lost to follow up<br><br>**Mean age:** 17 to 40 years<br><br>**Inclusion criteria:** Participants were women aged 17 to 40 years, married, in their third trimester of pregnancy, and intending to reside in the study area for the duration of the study.<br><br>**Exclusion criteria:** women with diagnosed serious medical/psychiatric condition requiring treatment, pregnancy-related illness (except for common conditions, such as anaemia), and substantial physical/learning disability	Intervention (n = 224)<br><br>cognitive-behavioral counselling<br><br>Mothers in the intervention group received 7 sessions of cognitive-behavioral counselling from antenatal to 6 months postpartum,<br><br>**Dosage:** The first session was delivered before birth,the second session immediately afterbirth, and the remaining 5 sessions monthly thereafter.<br><br>**Mode:** counselling sessions<br><br>**Given by:** LHWs<br><br>**Duration:** 12 months<br><br>Control (n = 230)<br><br>control group received an equal number of routine sessions	Breastfeeding initiation within 1 hour of birth (n)<br><br>Exclusive breastfeeding at 6 month (n)	**Study start date and end date:** May 2009 and April 2010<br><br>**Study duration:** 11 months<br><br>**Conflict of interest:** none to declare<br><br>**Source of funding:** This study was supported by PRIDE, Pakistan (Primary Health Care Revitalisation, Integration, and Decentralisation in Earthquake Affected Areas), a project funded by the US Agency for International Development
Stephenson 2017	**Complementary Feeding**<br><br>**Design:** Randomized controlled trial	**Location/Setting:** in 8 villages surrounding Limera in the Machinga District and 9 villages surrounding Masenjere in the Nsjane District of Southern Malawi<br><br>**Sample size:** 355 infants<br><br>**Dropouts/withdrawals:** 18/117, 21/120 and 25/118 were lost to follow up due to death, malnutrition or noncompliance. Reasons similar across groups<br><br>**Mean age:** 5.8 months<br><br>**Inclusion criteria:** All children aged 6 mo and living within walking distance of one of the village clusters were recruited<br><br>**Exclusion criteria:** Exclusion criteria included severe or moderate acute mal nutrition, receiving supplemental food from another intervention program, and having a chronic non-infectious disease or a congenital abnormality.	Intervention Cowpea (n =117), Common bean (n =120)<br><br>The intervention groups received daily complementary food composed of either cowpeas (*Vigna unguiculata*)or common beans (*Phaseolus vulgaris*);<br><br>**Mode:** complementary food supplements<br><br>**Given by:** mothers and care givers<br><br>**Duration:** 6 months<br><br>Control (n = 118)<br><br>the control group received a corn-soy blend flour throughout the 24 wk of study.	Height-for-age Z scores	**Study start date and end date:** between July 2015 and October 2016<br><br>**Study duration:** 16 months<br><br>**Conflict of interest:** none to declare<br><br>**Source of funding:** US Agency for International Development (USAID), as part of Feed the Future, the US government's global hunger and food security initiative
Susin 2008	**Exclusive Breastfeeding**<br><br>**Design:** Randomized controlled trial	**Location/Setting:** in a maternity ward of Hospital de Clínicas de Porto Alegre, Brazil<br><br>**Sample size:**586 mother-father-infant triads<br><br>**Dropouts/withdrawals:** 547 triads completed the study<br><br>**Mean age:** not **specified**	Intervention<br><br>Group1 (n = 193)<br><br>intervention with both mothers and fathers.<br><br>Group 2 (n = 192)<br><br>intervention with mothers only	Breastfeeding 4 months (n)<br><br>Exclusive breastfeeding for 6 months (n)	**Study start date and end date:** not **specified**<br><br>**Study duration:** not **specified**<br><br>**Conflict of interest:** not **specified**<br><br>**Source of funding:** not **specified**

		**Inclusion criteria:** couples living together in the city of Porto Alegre who had infants born with no health problems and birth weight equal to or greater than 2500 g and who initiated breastfeeding.<br><br>**Exclusion criteria:** Triads whose parents separated during follow-up were excluded from the study	**Dosage:** Visited infants' 1st, 2nd, 4th, and 6th months of life or until breastfeeding was interrupted, if this occurred before the end of the sixth month.<br><br>**Mode:** home visits<br><br>**Given by:** trained paediatrician.<br><br>**Duration:** 6 months<br><br>Control: not exposed to the intervention<br><br>(n = 201)		
Tahir 2013	**Exclusive Breastfeeding**<br><br>**Design:** Randomized controlled trial	**Location/Setting:** in Maternity wards in a public hospital in Kuala Lumpur, Malaysia.<br><br>**Sample size:** 357 mothers<br><br>**Dropouts/withdrawals:** 19/179 from intervention group and 20/178 from control group<br><br>**Mean age:** 28.58 years<br><br>**Inclusion criteria:** the women were required to be 18 years of age or older and of Malaysian nationality. Each mother must have delivered a single infant at 37 or more weeks of gestation. Further requirements for participation in the study included an intention to breast feed and the ability to understand and communicate in spoken Malay or English.<br><br>**Exclusion criteria:** Women with multiple pregnancies or medical problems that might hinder breastfeeding, women that delivered via Caesarean section,or women whose baby subsequently required prolonged care in a Special Care Nursery were not eligible	Intervention (n = 179)<br><br>Telephone lactation counselling twice monthly in addition to receiving the current conventional care of postnatal breastfeeding support.<br><br>**Mode:** Telephone counselling<br><br>**Given by:** certified lactation counsellors<br><br>**Duration:** 6 months<br><br>Control (n = 178)	Exclusive breastfeeding for 1 month and 6 months (n)	**Study start date and end date:** April 2010 to February 2011<br><br>**Study duration:** 11 months<br><br>**Conflict of interest:** none to declare<br><br>**Source of funding:** received funding from the Institute of Research<br><br>Management and Consultancy, University of Malaya
Thakur 2012	**Complementary Feeding**<br><br>**Design:** Randomized controlled trial	**Location/Setting:** in Maternal Care and Health Training Institute (Azimpur, Dhaka) and Dhaka Medical College Hospital (Dhaka) in Bangladesh<br><br>**Sample size:** 184 infants<br><br>**Dropouts/withdrawals:** no lost to follow up is noted<br><br>**Mean age:** mothers baseline age was 22.4 years<br><br>**Inclusion criteria:** mothers who attended the Maternal Care and Health Training Instituteand Dhaka Medical College Hospit for expected delivery<br><br>**Exclusion criteria:** Exclusion criteria considered were women having caesarian section, retained placenta,multiple births, babies who were born at night after 2100 h,and physically (disabled, wounded) and mentally (shocked,disturbed, etc. as stated by attending family members) handicapped.	Intervention (n = 92)<br><br>nutrition education twice a month for 2 months after delivery<br><br>**Mode:** education sessions<br><br>**Given by:** not **specified**<br><br>**Duration:** 2 months<br><br>Control (n = 92)	Breastfeeding in 1 hour (n)<br><br>Exclusive breastfeeding for 2 months (n)<br><br>Height gain after 1 month and 2 months (cm)<br><br>Infection in 1 month (n)<br><br>Diarrhea in 2 months (n)	**Study start date and end date:** not **specified**<br><br>**Study duration:** not **specified**<br><br>**Conflict of interest:** none to declare<br><br>**Source of funding:** not **specified**
Tomedi 2012	**Complementary Feeding**<br><br>**Design:** Quasi randomized trial	**Location/Setting:** in Rural villages in the arid lands of eastern Kenya with a high prevalence of child malnutrition<br><br>**Sample size:** 269 infants<br><br>**Dropouts/withdrawals:** 9/120 lost from intervention group and 6/149 lost from control group<br><br>**Mean age:** 13.6 months<br><br>**Inclusion criteria:** CHW identified every child aged 6–20 months in their village<br><br>**Exclusion criteria:** not **specified**	Intervention (n = 120)<br><br>monthly food ration for the index child, a separate family ration, and group education on complementary feeding and hygiene given monthly<br><br>**Mode:** monthly food ration<br><br>**Given by:** researchers<br><br>**Duration:** 7 months<br><br>Control (n = 149)	stunting prevalence (n)<br><br>Weight for age Z score<br><br>Infection (n)<br><br>Diarrhea (n)	**Study start date and end date:** November 2009 to March 2010<br><br>**Study duration:** 5 months<br><br>**Conflict of interest:** none to declare<br><br>**Source of funding:** study was supported by a University of New Mexico School of Medicine Research Allocation Committee Grant as well as funds from Global Health Partnerships.
Tylleskar 2011	**Exclusive Breastfeeding**<br><br>**Design:** Randomized controlled trial	**Location/Setting:** in 24 communities in Burkina Faso, 24 in Uganda, and 34 in South Africa<br><br>**Sample size:** 2579 mother–infant pairs were assigned to the intervention or control clusters in Burkina Faso (n=392 and n=402, respectively), Uganda (n=396 and n=369, respectively), and South Africa (n=535 and 485, respectively)<br><br>**Dropouts/withdrawals:** Minimal lost to follow up from the 3 sites was noted<br><br>**Mean age:** not **specified**<br><br>**Inclusion criteria:** The pre inclusion criteria were that the woman resided in the selected cluster; was 7 months or visibly pregnant; had no plans to move in the forthcoming year; and provided informed consent<br><br>**Exclusion criteria:** severe psychological illness, which could interfere with consent and study participation; giving birth more than 1 week before pre inclusion; or a plan to replacement feed.	2579 mother–infant pairs were assigned to the intervention or control clusters in Burkina Faso (n = 392 and n = 402, respectively), Uganda (n = 396 and n = 369, respectively), and South Africa (n = 535 and 485, respectively)<br><br>1 antenatal breastfeeding peer counselling visit and four post-delivery visits by trained peers. 5 visits on in the 3rd trimester.<br><br>In Burkina Faso, mothers were scheduled to have home visits during the 1st week postnatally, and thereafter at weeks 2, 4, 8, 16, and 20.<br><br>In Uganda and South Africa, home visits were scheduled within the 1st week and thereafter at weeks 4, 7, and 10.<br><br>**Mode:** home visits<br><br>**Given by:** peer counsellors<br><br>**Duration:** 10 weeks<br><br>Control: no intervention	Exclusive breastfeeding at 12 weeks (n)	**Study start date and end date:** not **specified**<br><br>**Study duration:** not **specified**<br><br>**Conflict of interest:** none to declare<br><br>**Source of funding:** European Union Sixth Framework International Cooperation–Developing Countries, Research Council of Norway<br><br>, Swedish International Development Cooperation Agency, Norwegian Programme for Development, Research and Education, South African National Research Foundation, and Rockefeller Brothers Foundation.
Vazir 2013	**Complementary Feeding** education<br><br>**Design:** Randomized controlled trial	**Location/Setting:** in Sixty villages in Andhra Pradesh, India<br><br>**Sample size:** 600 infant-mother dyads	Intervention<br><br>Group 1 (n = 200)	Change in length (cm) and weight (kg)<br><br>Prevalence of stunting (n)	**Study start date and end date:** not **specified**<br><br>**Study duration:** 1 year

		**Dropouts/withdrawals:** RCF&PG (22%) compared to the CG (9%) and CFG (16%)<br><br>**Mean age:** 3–15 months of age<br><br>**Inclusion criteria:** explained the study objectives to all the pregnant women in the villages and asked ifthey would like to participate in the study. There were no refusals<br><br>**Exclusion criteria:** not **specified**	the Complementary Feeding Group (CFG) received the ICDS and the World Health Organization recommendations on breastfeeding (x2/mo) and complementary foods (x4 month)<br><br>Group 2 (n = 200)<br><br>Responsive Complementary Feeding & Play Group (RCF&PG) received the same intervention as the CFG plus skills for responsive feeding and psychosocial stimulation.<br><br>**Dosage:** biweekly<br><br>**Mode:** education<br><br>**Given by:** Trained Village Women (VW)<br><br>**Duration:** 12 months<br><br>Control (n = 200)<br><br>Control Group (CG), received routine Integrated Child Development Services (ICDS)	Hemoglobin levels (g/dl)	**Conflict of interest:** not **specified**<br><br>**Source of funding:** Indian Council of Medical Research, India and UNICEF, New York.
Vitolo 2005	**Complementary Feeding** education<br><br>**Design:** Randomized controlled trial	**Location/Setting:** in Centennial Hospital , the city of São Leopoldo, Rio Grande do Sul, Brazil<br><br>**Sample size:** 469 newborns<br><br>**Dropouts/withdrawals:** 34/197 from intervention group and 38/272 lost to follow up from control group<br><br>**Mean age:** age greater than 37 weeks.<br><br>**Inclusion criteria:** birth weight greater than 2,500 g, age greater than 37 weeks.<br><br>**Exclusion criteria:** HIV-positive mothers, need for the intensive care unit, twins, congenital malformation	Intervention (n = 197)<br><br>Parents of the intervention group received nutritional orientation during the child's first year of life 10 home visits, performed within the first 10 days after optometry and then monthly up to 6 months, at 8, 10 and 12 months.<br><br>**Mode:** education sessions<br><br>**Given by:** field workers<br><br>**Duration:** 12 months<br><br>Control (n = 272)	Exclusive breastfeeding at 1 month and at 6 months (n)<br><br>Diarrhea (n)	**Study start date and end date:** not **specified**<br><br>**Study duration:** not **specified**<br><br>**Conflict of interest:** none to declare<br><br>**Source of funding:** This study received financial support from the National Council for Scientific and Technological Development (CNPq).
Younes 2015	Breastfeeding intervention<br><br>**Design:** Quasi randomized trial	**Location/Setting:** in three districts, Bogra, Faridpur and Moulavi bazar in Bangladesh<br><br>**Sample size:** 1897 infants<br><br>**Dropouts/withdrawals:** increase in participants in the final analysis. It is a controlled before and after study.<br><br>**Mean age:** 30.75 months<br><br>**Inclusion criteria:** Women were eligible to become members of the women's groups if they were 15–49 years of age and resided in the inter-vention areas.<br><br>**Exclusion criteria:** not **specified**	Intervention (n = 926)<br><br>women support groups<br><br>**Mode:** support groups<br><br>**Given by:** facilitators<br><br>**Duration:** 12 months<br><br>Control (n = 971)	Exclusive breastfeeding at 6 months (n)<br><br>Infection (n)<br><br>Diarrhea at 2 weeks(n)	**Study start date and end date:** April 2010 to December 2011<br><br>**Study duration:** 21 months<br><br>**Conflict of interest:** none to declare<br><br>**Source of funding:** The Big Lottery Fund (UK)
Zaman 2008	**Complementary Feeding**<br><br>**Design:** Randomized controlled trial	**Location/Setting:** in 60 health care centres operated by the Directorate of Health and the Lahore Metropolitan Corporation (LMC) in Lahore, Pakistan<br><br>**Sample size:** 320 infant mother pairs<br><br>**Dropouts/withdrawals:** 31/151 and 32/169 pairs were lost to follow up due to non availiability and relocation. Reasons similar across groups<br><br>**Mean age:** not **specified** separately<br><br>**Inclusion criteria:** The first 10 children aged 6-18 months, coming for consultation at each health centre, were selected.<br><br>**Exclusion criteria:** Any child reporting with illness for which referral was required was ex-cluded. Any child not living in the study area orwho was reportedly expected to move away during the following six months was also excluded	Intervention (n = 151 pairs)<br><br>Mother-child pairs were visited at home within two weeks,45 days, and 180 days after recruitment.<br><br>**Mode:** education<br><br>**Given by:** health workers<br><br>**Duration:** 6 months<br><br>Control (n = 169 pairs)	Height-for-age Z-scores<br><br>Weight-for- height Z-scores<br><br>Weight for age Z-scores	**Study start date and end date:** not **specified**<br><br>**Study duration:** 6 months<br><br>**Conflict of interest:** not **specified**<br><br>**Source of funding:** Department of Child and Adolescent Health of WHO
Zhang 2016	**Complementary Feeding**<br><br>**Design:** Randomized controlled trial	**Location/Setting:** in one intervention county and one control county in rural Qinghai Province, China<br><br>**Sample size:** 2605<br><br>**Dropouts/withdrawals:** The baseline, mid-term and end-line surveys were conducted on 1804, 2187 and 2186 children aged 6–23 months in the intervention county in August 2012, 2013 and 2014, respectively, and 804, 680 and 790 children in the control county<br><br>**Mean age:** 14.5 months<br><br>**Inclusion criteria:** (1) children aged between 6 and 23 months; (2)primary caregivers; and (3) rural children who could be distinguished by their urban or rural registration(known as Hukou in China), place of registration, and geographic location<br><br>**Exclusion criteria:** (1) children with a structural or genetic birth defect, such as neural tube defects, congenital heart disease or phenylketonuria; (2) caregivers who refused to participate.	Intervention (n = 1801)<br><br>Complementary food supplements (containing protein, fat, carbohydrate, vitamin A, B1, B2, B12, D3, folic acid, iron, zinc and calcium) with complementary feeding counselling given daily<br><br>**Mode:** food supplement sachets with counselling<br><br>**Given by:** Village doctors<br><br>**Duration:** 24 months<br><br>Control (n = 804)	Stunting (n)	**Study start date and end date:** 2012 to 2014<br><br>**Study duration:** 2 years<br><br>**Conflict of interest:** none to declare<br><br>**Source of funding:** United Nations Children's Fund (UNICEF)

Table S7: Summary of Findings Table of Breastfeeding Education Interventions

Certainty assessment							№ of patients		Effect		Certainty	Importance
№ of studies	Study design	Risk of bias	Inconsistency	Indirectness	Imprecision	Other considerations	Breastfeeding Education Intervention	Control	Relative (95% CI)	Absolute (95% CI)		
**Early initiation of breastfeeding**												
14	randomised trials	not serious	very serious [a]	not serious	not serious	none	32156/49816 (64.5%)	16190/34276 (47.2%)	**RR 1.20** (1.12 to 1.28)	**94 more per 1,000** (from 57 more to 132 more)	⊕⊕◯◯ LOW	
**Exclusive breastfeeding at 3 months**												
6	randomised trials	very serious [b]	serious [c]	not serious	not serious	none	1243/2144 (58.0%)	558/1919 (29.1%)	**RR 2.02** (1.88 to 2.17)	**297 more per 1,000** (from 256 more to 340 more)	⊕◯◯◯ VERY LOW	
**Exclusive breastfeeding at 6 months**												
19	randomised trials	very serious [d]	very serious [e]	not serious	not serious	none	3716/7057 (52.7%)	2574/6869 (37.5%)	**RR 1.53** (1.47 to 1.58)	**199 more per 1,000** (from 176 more to 217 more)	⊕◯◯◯ VERY LOW	

a. Highly heterogeneous data ($Chi^2 = 243.17$; ($P < 0.00001$); $I^2 = 94\%$)

b. Major risk of bias detected across multiple studies; i.e. high risk random sequence generation in one study, high risk allocation concealment in three studies, high risk of performance bias in two studies, high risk detection bias in two studies, high risk of attrition bias in one study and high risk of other biases in one study; out of six studies.

c. Moderately heterogeneous data ($Chi^2 = 35.63$; $I^2 = 78\%$)

d. Major risk of bias detected across multiple studies; i.e. high risk random sequence generation in two studies, high risk allocation concealment in two studies, high risk of performance bias in seven studies, high risk of detective bias in four studies, high risk of attrition bias in four studies and high risk of other biases four studies; out of 19 studies.

e. Highly heterogeneous data ($Chi^2 = 423.55$; ($P < 0.00001$); $I^2 = 94\%$)

Table S8: Summary of Findings Table of Complementary Education Interventions

Certainty assessment							№ of patients		Effect		Certainty	Importance
№ of studies	Study design	Risk of bias	Inconsistency	Indirectness	Imprecision	Other considerations	Complementary Feeding Interventions	Control	Relative (95% CI)	Absolute (95% CI)		
**Weight-for-age (z-scores)**												
13	randomised trials	serious [a]	very serious [b]	serious [c]	not serious	none	2310	2233	-	MD **0.13 higher** (0.02 lower to 0.28 higher)	⊕◯◯◯ VERY LOW	
**Height-for-age (z-scores)**												
14	randomised trials	not serious	not serious	serious [c]	not serious	none	5947	3496	-	MD **0.12 higher** (0.05 higher to 0.19 higher)	⊕⊕⊕◯ MODERATE	
**Weight-for-height (z-scores)**												
12	randomised trials	not serious	very serious [d]	serious [c]	not serious	none	5499	6877	-	MD **0.02 higher** (0.01 lower to 0.04 higher)	⊕◯◯◯ VERY LOW	
**Stunting**												
12	randomised trials	not serious	serious [e]	serious [c]	not serious	publication bias strongly suspected [f]	1153/8021 (14.4%)	1217/7981 (15.2%)	**RR 0.87** (0.77 to 0.98)	**20 fewer per 1,000** (from 35 fewer to 3 fewer)	⊕◯◯◯ VERY LOW	
**Wasting (prevalence)**												
7	randomised trials	not serious	not serious	serious [c]	not serious	none	1676/5188 (32.3%)	2503/6649 (37.6%)	**RR 0.89** (0.80 to 0.99)	**41 fewer per 1,000** (from 75 fewer to 4 fewer)	⊕⊕⊕◯ MODERATE	

a. High risk of bias detected across multiple studies; high risk random sequence generation in three studies, high risk allocation concealment in one study, high risk of performance bias in one study, high risk of detection bias in two studies, high risk of attrition bias in three studies and high risk of other biases in one study; out of 13 studies.

b. Highly heterogeneous data ($Chi^2 = 112.51$; ($P < 0.00001$); $I^2 = 87\%$)

c. Complementary feeding is being compared alongside complementary feeding education.

d. Highly heterogeneous data (Chi² = 8334.56; (P < 0.00001); I² = 100%)

e. Moderately heterogeneous data (Chi² = 60.47; (P < 0.00001); I² = 72%)

f. Asymmetry noted upon inspection of funnel plot

Table S9: Summary of Findings Table of Supplementary Feeding Educations

Certainty assessment							№ of patients		Effect		Certainty	Importance
№ of studies	Study design	Risk of bias	Inconsistency	Indirectness	Imprecision	Other considerations	Supplementary Feeding Interventions	Control	Relative (95% CI)	Absolute (95% CI)		
**Height-for-age (z-scores)**												
3	randomised trials	serious [a]	very serious [b]	not serious	not serious	none	2610	957	-	MD **0.05 higher** (0.32 lower to 0.43 higher)	⊕◯◯◯ VERY LOW	
**Weight-for-age (z-scores)**												
3	randomised trials	serious [a]	very serious [c]	not serious	not serious	none	2610	957	-	MD **0.44 higher** (0.03 lower to 0.92 higher)	⊕◯◯◯ VERY LOW	
**Weight-for-height (z-scores)**												
3	randomised trials	serious [a]	not serious	not serious	not serious	none	2610	957	-	MD **0.1 higher** (0.1 lower to 0.3 higher)	⊕⊕⊕◯ MODERATE	
**Stunting**												
5	randomised trials	not serious	very serious [d]	not serious	not serious	none	809/3307 (24.5%)	395/1425 (27.7%)	**RR 1.13** (0.73 to 1.74)	**36 more per 1,000** (from 75 fewer to 205 more)	⊕⊕◯◯ LOW	
**Wasting**												
5	randomised trials	not serious	very serious [e]	not serious	not serious	none	830/4799 (17.3%)	673/2720 (24.7%)	**RR 0.75** (0.55 to 1.02)	**62 fewer per 1,000** (from 111 fewer to 5 more)	⊕⊕◯◯ LOW	

a. High risk of bias detected in few studies; high risk of random sequence generation in one study and high risk of allocation concealment in the same study; out of three studies.

b. Highly heterogeneous data (Chi² = 88.68; (P < 0.00001); I² = 98%)

c. Highly heterogeneous data (Chi² = 172.9; (P < 0.00001); I² = 99%)

d. Highly heterogeneous data (Chi² = 37.72; (P < 0.00001); I² = 87%)

e. Highly heterogeneous data (Chi² = 25.65; (P < 0.0001); I² = 84%)

1. Aksu H, Küçük M, G D. The effect of postnatal breastfeeding education/support offered at home 3 days after delivery on breastfeeding duration and knowledge: a randomized trial. The Journal of Maternal-Fetal & Neonatal Medicine. 2011;24(2):354-61.
2. Albernaz E, Victora CG, Haisma H, Wright A, WA C. Lactation counseling increases breastfeeding duration but not breast milk intake as measured by isotopic methods. The Journal of nutrition. 2003;133(1):205-10.
3. Baqui AH, El-Arifeen S, Darmstadt GL, Ahmed S, Williams EK, Seraji HR, et al. Effect of community-based newborn-care intervention package implemented through two service-delivery strategies in Sylhet district, Bangladesh: a cluster-randomised controlled trial. The lancet. 2008;371(9628):1936-44.
4. Cangöl E, NH. Ş. The Effect of a Breastfeeding Motivation Program Maintained During Pregnancy on Supporting Breastfeeding: A Randomized Controlled Trial. Breastfeeding Medicine. 2017;12(4):218-26.
5. Flax VL, Negerie M, Ibrahim AU, Leatherman S, Daza EJ, ME. B. Integrating Group Counseling, Cell Phone Messaging, and Participant-Generated Songs and Dramas into a Microcredit Program Increases Nigerian Women's Adherence to International Breastfeeding Recommendations–3. The Journal of nutrition. 2014;144(7):1120-4.
6. Hanson C, Manzi F, Mkumbo E, Shirima K, Penfold S, Hill Z, et al. Effectiveness of a home-based counselling strategy on neonatal care and survival: a cluster-randomised trial in six districts of rural southern Tanzania. PLoS medicine. 2015;12(9):e1001881.
7. Ijumba P, Doherty T, Jackson D, Tomlinson M, Sanders D, Swanevelder S PL. Effect of an integrated community-based package for maternal and newborn care on feeding patterns during the first 12 weeks of life: a cluster-randomized trial in a South African township. Public health nutrition. 2015;18(14):2660-8.
8. Jahan K, Roy SK, Israt S, Ferdouse K, SB S. Impact of nutrition education on pregnancy weight gain and birth outcome. Annals of Nutrition & Metabolism. 2013;63(Supplement):756.
9. Kirkwood BR, Manu A, Asbroek AH, Soremekun S, Weobong B, T G. Effect of the Newhints home visits intervention on neonatal mortality rate and care practices in Ghana: a cluster randomised controlled trial. Lancet. 2013;381(9884):2184–92.
10. Mangwi Ayiasi R, Kolsteren P, Batwala V, Criel B, Orach CG. Effect of Village Health Team Home Visits and Mobile Phone Consultations on Maternal and Newborn Care Practices in Masindi and Kiryandongo, Uganda: A Community-Intervention Trial. PLoS One. 2016;11(4):e0153051.
11. Penfold S, Manzi F, Mkumbo E, Temu S, Jaribu J, Shamba DD, et al. Effect of home-based counselling on newborn care practices in southern Tanzania one year after implementation: a cluster-randomised controlled trial. BMC pediatrics. 2014;14(1):187.
12. Raeisi K, Shariat M, Nayeri F, Raji F, H D. A single center study of the effects of trained fathers' participation in constant breastfeeding. Acta Medica Iranica. 2014;52(9):694-6.
13. Sikander S, Maselko J, Zafar S, Haq Z, Ahmad I, Ahmad M, et al. Cognitive-behavioral counselling for exclusive breastfeeding in rural pediatrics: a cluster RCT. Pediatrics. 2015;135(2):e424-31.
14. Thakur SK, Roy SK, Paul K, Khanam M, Khatun W, D S. Effect of nutrition education on exclusive breastfeeding for nutritional outcome of low birth weight babies. European journal of clinical nutrition. 2012;66(3):376.
15. Khan AI, Hawkesworth S, Ekström EC, Arifeen S, Moore SE, Frongillo EA, et al. Effects of exclusive breastfeeding intervention on child growth and body composition: the MINIMat trial, Bangladesh. Acta Paediatrica. 2013;102(8):815-23.
16. AH A. Breastfeeding preterm infants: an educational program to support mothers of preterm infants in Cairo, Egypt. Pediatric Nursing. 2008;34(2):125-30.
17. Bhandari N, Bahl R, Mazumdar S, Martines J, Black RE, Bhan MK. Effect of community-based promotion of exclusive breastfeeding on diarrhoeal illness and growth: a cluster randomised controlled trial. The Lancet. 2003;361(9367):1418-23.
18. Froozani MD, Permehzadeh K, Motlagh AR, B. G. Effect of breastfeeding education on the feeding pattern and health of infants in their first 4 months in the Islamic Republic of Iran. Bulletin of the World Health Organization. 1999;77(5):381–5.
19. Gu Y, Zhu Y, Zhang Z, H W. Effectiveness of a theory-based breastfeeding promotion intervention on exclusive breastfeeding in China: A randomised controlled trial. Midwifery. 2016;42:93-9.
20. Morrow AL, Guerrero ML, Shults J, Calva JJ, Lutter C, J B. Efficacy of home-based peer counselling to promote exclusive breastfeeding: a randomized controlled trial. Lancet. 1999;353(9160):1226–31.
21. Tylleskar T, Jackson D, Meda N, Engebretsen IM, ChopraM, AH D. Exclusive breastfeeding promotion by peer counsellors in sub-Saharan Africa (PROMISE-EBF): a cluster-randomised trial. Lancet. 2011;378(9789):420–7.
22. Agrasada GV, Ewald U, Kylberg E, Gustafsson J. Exclusive breastfeeding of low birth weight infants for the first six months: infant morbidity and maternal and infant anthropometry. Asia Pac J Clin Nutr. 2011;20(1):62-8.
23. Aidam BA, Perez-Escamilla R, A. L. Lactation counseling increases exclusive breastfeeding rates in Ghana. Journal of Nutrition. 2005;135(7):1691-5.

24. Arifeen SE, Hoque DE, Akter T, Rahman M, Hoque ME, Begum K, et al. Effect of the Integrated Management of Childhood Illness strategy on childhood mortality and nutrition in a rural area in Bangladesh: a cluster randomised trial. The Lancet. 2009;1;374(9687):393-403.
25. Bolam A, Manandhar DS, Shrestha P, Ellis M AM. The effects of postnatal health education for mothers on infant care and family planning practices in Nepal: a randomised controlled trial. BMJ. 1998;316(7134):805-11.
26. Khresheh R, Suhaimat A, Jalamdeh F, L. B. The effect of a postnatal education and support program on breastfeeding among primiparous women: a randomized controlled trial. International journal of nursing studies. 2011;48(9):1058-65.
27. Kimani-Murage EW, Griffiths PL, Wekesah FM, Wanjohi M, Muhia N, Muriuki P, et al. Effectiveness of home-based nutritional counselling and support on exclusive breastfeeding in urban poor settings in Nairobi: a cluster randomized controlled trial. Globalization and health. 2017;13(1):90.
28. Kupratakul J, Taneepanichskul S, Voramongkol N, V P. A randomized controlled trial of knowledge sharing practice with empowerment strategies in pregnant women to improve exclusive breastfeeding during the first six months postpartum. J Med Assoc. 2010;93(9):1009-8.
29. Ochola SA, Labadarios D, RW N. Impact of counselling on exclusive breast-feeding practices in a poor urban setting in Kenya: a randomized controlled trial. Public health nutrition. 2013;16(10):1732-40.
30. Susin LR, ER G. Inclusion of fathers in an intervention to promote breastfeeding: impact on breastfeeding rates. Journal of Human Lactation. 2008;24(4):386–92.
31. Tahir NM, N A-S. Does telephone lactation counselling improve breastfeeding practices?: A randomised controlled trial. International journal of nursing studies. 2013;50(1):16-25.
32. Vitolo MR, Bortolini GA, Feldens CA, MD D. Impacts of the 10 Steps to Healthy Feeding in Infants: a randomized field trial. Cadernos de saude publica. 2005;21(5):1448-57.
33. Younes L, Houweling TA, Azad K, Kuddus A, Shaha S, Haq B, et al. The effect of participatory women's groups on infant feeding and child health knowledge, behaviour and outcomes in rural Bangladesh: a controlled before-and-after study. J Epidemiol Community Health. 2015;69(4):374-81.
34. Fadnes LT, Nankabirwa V, Engebretsen IM, Sommerfelt H, Birungi N, Lombard C, et al. Effects of an exclusive breastfeeding intervention for six months on growth patterns of 4–5 year old children in Uganda: the cluster-randomised PROMISE EBF trial. BMC public health. 2016;16(1):555.
35. Nair N, Tripathy P, Sachdev HS, Pradhan H, Bhattacharyya S, Gope R, et al. Effect of participatory women's groups and counselling through home visits on children's linear growth in rural eastern India (CARING trial): a cluster-randomised controlled trial. The Lancet Global Health. 2017;5(10):e1004-16.
36. Navarro JI, Sigulem DM, Ferraro AA, Polanco JJ, AJ B. The double task of preventing malnutrition and overweight: a quasi-experimental community-based trial. BMC Public Health. 2013;13(1):212.
37. Schwartz R, Vigo A, de Oliveira LD, ERJ G. The effect of a pro-breastfeeding and healthy complementary feeding intervention targeting adolescent mothers and grandmothers on growth and prevalence of overweight of preschool children. PLOS One. 2015;10(7):e0131884.
38. More NS, Bapat U, Das S, Alcock G, Patil S, Porel M, et al. Community mobilization in Mumbai slums to improve perinatal care and outcomes: a cluster randomized controlled trial. PLoS medicine. 2012;9(7):e1001257.
39. Bauserman M, Lokangaka A, Gado J, Close K, Wallace D, Kodondi KK, et al. A cluster-randomized trial determining the efficacy of caterpillar cereal as a locally available and sustainable complementary food to prevent stunting and anaemia. Public health nutrition. 2015;Jul 18(10):1785-92.
40. Guldan GS, Fan HC, Ma X, Ni ZZ, Xiang X, MZ T. Culturally appropriate nutrition education improves infant feeding and growth in rural Sichuan, China. J Nutr. 2000;130(5):1204-11.
41. Iannotti LL, Lutter CK, Stewart CP, Gallegos Riofrío CA, Malo C, Reinhart G, et al. Eggs in Early Complementary Feeding and Child Growth: A Randomized Controlled Trial. Pediatrics. 2017;140(1):e20163459.
42. Lutter CK, Rodriguez A, Fuenmayor G, Avila L, Sempertegui F, J E. Growth and micronutrient status in children receiving a fortified complementary food. J Nutr. 2008;138(2):379-88.
43. Mangani C, Maleta K, Phuka J, Cheung YB, Thakwalakwa C, Dewey K, et al. Effect of complementary feeding with lipid-based nutrient supplements and corn–soy blend on the incidence of stunting and linear growth among 6-to 18-month-old infants and children in rural Malawi. Maternal & child nutrition. 2015;132-43:132-43.
44. Oelofse A, Van Raaij JMA, Benade AJS, Dhansay MA, Tolboom JJM, J H. The effect of a micronutrient-fortified complementary food on micronutrient status, growth and development of 6- to 12-month-old disadvantaged urban South African infants. Int J Food Sci Nutr. 2003;54:399-407.
45. Olaya GA, Lawson M, MS F. Efficacy and safety of new complementary feeding guidelines with an emphasis on red meat consumption: a randomized trial in Bogota, Colombia. The American journal of clinical nutrition. 2013;98(4):983-93.
46. Penny ME, Creed-Kanashiro HM, Robert RC, Narro MR, Caulfield LE, RE B. Effectiveness of an educational intervention delivered through the health services to improve nutrition in young children: a cluster-randomised controlled trial. Lancet. 2005;365(9474):1863–72.
47. Roy SK, Jolly SP, S S. Prevention of malnutrition among young children in rural Bangladesh by a food-health-care educational intervention: a randomized, controlled trial. Food Nutr Bulletin. 2007;28(4):375–83.
48. Santos I, Gigante Denise P, Coitinho Denise C, VNCJ HH. Evaluation of the impact of a nutritional programme for undernourished children in Brazil. Cad Saude Publica. 2005;21(3):776–85.
49. Santos I, Victora CG, J M. Nutrition counselling increases weight gain among Brazilian children. 2001;131(11):2866–73.
50. Stephenson KB, Agapova SE, Divala O, Kaimila Y, Maleta KM, Thakwalakwa C, et al. Complementary feeding with cowpea reduces growth faltering in rural Malawian infants: A blind, randomized controlled clinical trial. The American journal of clinical nutrition. 2017;106(6):1500-7.
51. Zaman S, Ashraf RN, J M. Training in complementary feeding counselling of healthcare workers and its influence on maternal behaviours and child growth: a cluster-randomized controlled trial in Lahore, Pakistan. J Health Popul Nutr. 2008;26(2):210.
52. Christian P, Shaikh S, Shamim AA, Mehra S, Wu L, Mitra M, et al. Effect of fortified complementary food supplementation on child growth in rural Bangladesh: a cluster-randomized trial. International journal of epidemiology. 2015;Aug 14;44(6):1862-76.
53. Martinez B, Webb MF, Gonzalez A, Douglas K, del Pilar Grazioso M, P R. Complementary feeding intervention on stunted Guatemalan children: a randomised controlled trial. BMJ paediatrics open. 2018;2:1.
54. Bhandari N, Bahl R, Nayyar B, Khokhar P, Rohde JE, MK. B. Food supplementation with encouragement to feed it to infants from 4 to 12 months of age has a small impact on weight gain. J Nutr. 2001;131(7):1946–51.
55. Bhandari N, Mazumder S, Bahl R, Martines J, Black RE, MK. B. An educational intervention to promote appropriate complementary feeding practices and physical growth in infants and young children in rural Haryana, India. J Nutr. 2004;134(9):2342–8.
56. Saleem AF, Mahmud S, Baig-Ansari N, AK. Z. Impact of maternal education about complementary feeding on their infants' nutritional outcomes in low-and middle-income households: a community-based randomized interventional study in Karachi, Pakistan. Journal of health, population, and nutrition. 2014;32(4):623.
57. Vazir S, Engle P, Balakrishna N, Griffiths PL, Johnson SL, Creed-Kanashiro H, et al. Cluster-randomized trial on complementary and responsive feeding education to caregivers found improved dietary intake, growth and development among rural Indian toddlers. Maternal & child nutrition. 2013;9(1):99-117.
58. Zhang Y, Wu Q, Wang W, van Velthoven MH, Chang S, Han H, et al. Effectiveness of complementary food supplements and dietary counselling on anaemia and stunting in children aged 6–23 months in poor areas of Qinghai Province, China: a controlled interventional study. BMJ open. 2016;6(10):e011234.
59. Isanaka S, Nombela N, Djibo A, Poupard M, Van Beckhoven D, Gaboulaud V, et al. Effect of preventive supplementation with ready-to-use therapeutic food on the nutritional status, mortality, and morbidity of children aged 6 to 60 months in Niger: a cluster randomized trial. JAMA. 2009;Jan 21;301(3):277-85.
60. Shi L, Zhang J, Wang Y, Caulfield LE, B G. Effectiveness of an educational intervention on complementary feeding practices and growth in rural China: a cluster randomised controlled trial. Public Health Nutr. 2009;13(04):556–65.
61. Hess SY, Bado L, Aaron GJ, Ouedraogo JB, Zeilani M, KH B. Maternal Child Nutr. 2011;7(4):357–67.
62. Schroeder DG, Pachón H, Dearden KA, Kwon CB, Ha TT, TT L. An integrated child nutrition intervention improved growth of younger, more malnourished children in northern Vietnam. Food and Nutrition Bulletin. 2002;23:4.
63. Tomedi A, Rohan-Minjares F, McCalmont K, Ashton R, Opiyo R, M M. Feasibility and effectiveness of supplementation with locally available foods in prevention of child malnutrition in Kenya. Public health nutrition. 2012;15(4):749-56.
64. de Oliveira LD, Giugliani ER, do Espírito Santo LC, Franca MC, Weigert EM, Kohler CV, et al. Effect of intervention to improve breastfeeding technique on the frequency of exclusive breastfeeding and lactation-related problems. Journal of Human Lactation. Journal of Human Lactation. 2006;22(3):315-21.
65. Grellety E, Shepherd S, Roederer T, Manzo ML, Doyon S, Ategbo EA, et al. Effect of mass supplementation with ready-to-use supplementary food during an anticipated nutritional emergency. PLoS One. 2012;Sep 12;7(9):e44549.
66. Kuusipalo H, Maleta K, Briend A, Manary M, P A. Growth and change in blood haemoglobin concentration among underweight Malawian infants receiving fortified spreads for 12 weeks: a preliminary trial. Journal of pediatric gastroenterology and nutrition. 2006;Oct 1;43(4):525-32.
